# Unequal harvests: AI-assisted evidence map of trends and gaps in global farmer health research along SDG 3 priorities

**DOI:** 10.1136/bmjopen-2025-110537

**Published:** 2026-06-01

**Authors:** Lena Jäggi, Irene Falgas Bague, Hannah Wey, Daniel Rüfli, Paola Gabriela Viglietti, Samuel Fuhrimann

**Affiliations:** 1 University of Basel, Basel, Switzerland; 2 Swiss Tropical and Public Health Institute, Allschwil, Switzerland; 3 Centre for Environmental and Occupational Health Research (CEOHR), School of Public Health, University of Cape Town, Rondebosch, South Africa; 4 ASCENT Laboratory, Psychology Department, University of Cape Town, Rondebosch, South Africa

**Keywords:** Review, EPIDEMIOLOGY, OCCUPATIONAL & INDUSTRIAL MEDICINE, Health Equity, PUBLIC HEALTH, Health policy

## Abstract

**Abstract:**

**Introduction:**

Ensuring the health of agricultural workers, the world’s largest labour force, is key for sustainable food production and progress towards the Sustainable Development Goals (SDGs).

**Methods:**

We conducted an artificial intelligence (AI)-assisted evidence map of research records on global farmer health published from 2015 to 2024. We searched bibliographic databases and screened titles/abstracts using SWIFT-Active Screener, a collaborative review platform that uses machine-learning prioritisation to rank records for human review. We retrieved 32 006 records. After manually screening 8533 records and stopping when the tool estimated ≥94% recall of relevant records, we included 1684 studies. We mapped research output by health topic category (non-communicable diseases (NCDs), communicable diseases, injuries and mental health) country income groups and alignment with SDG 3 targets.

**Results:**

Despite 98% of the agricultural workforce living in low- and middle-income countries (LMICs), 52% of studies originate from high-income countries (HICs). Research focuses on NCDs (29%) and injuries (26%), with LMICs focusing on pesticide poisoning and HIC on accidents. Mental health emerges as a key topic in HICs, with the proportion of publications nearly doubling from 2021 to 2024 but remains underexplored in LMICs. Key gaps with high relevance to farming populations and climate change, such as heat-related illnesses, occupational injuries and musculoskeletal conditions, are not well represented in SDG 3 indicators.

**Conclusion:**

Our findings highlight urgent needs for a more equitable and comprehensive global research agenda that integrates agricultural worker health into sustainability frameworks beyond the SDG era, ensuring the resilience and well-being of food producers worldwide.

**STRENGTHS AND LIMITATIONS OF THIS STUDY**

- Applied a rigorous scoping review methodology to screen titles and abstracts from over 30 000 research records from Ovid/MEDLINE and PsycInfo, two of the largest health research databases, in a timely way.
- Applied screening with SWIFT-Active Screener, which applies machine learning to prioritise relevant records, to speed up the review process while keeping accuracy through manual screening of over 8000 records, iterative training and team discussions.
- Grouped health outcomes through an iterative mapping process to capture major public health priorities across Global Burden of Disease categories and Sustainable Development Goals 3 indicators.
- Title and abstract screening were done by a single reviewer, which improved speed but differs from the standard two-reviewer method of systematic reviews.
- Only published studies in two databases were included, which may miss relevant evidence and under-represent informal or unpaid agricultural work.

## Introduction

 Agriculture employs approximately 26% of the global workforce according to International Labour Organization (ILO) estimates,^1 2^ but this figure masks striking disparities between high-income countries (HICs) and low- and middle-income countries (LMICs). In many HICs, fewer than 2% of workers are employed in agriculture, compared with over 60% in many LMICs. For instance, agriculture accounts for only 1.7% of the workforce in the USA, but employed an estimated 40.7% in India and over 80% in Burkina Faso.^2 3^ Furthermore, these numbers are likely an underestimation, since they do not fully account for the vast percentage of people who work in agriculture part-time. According to the World Bank, agriculture contributes 25% to the GDP (gross domestic product) in low-income countries, while it only contributes 1% in HICs.^3^

Farmers and agricultural workers are the largest occupational group globally, and are thus central to achieving multiple Sustainable Development Goals (SDGs).^4^ Farmers’ contributions to global development are critical. Their role in food production directly supports SDG 2 (Zero Hunger) and SDG 12 (Responsible Consumption and Production). Additionally, their working conditions and economic vulnerabilities intersect with SDG 1 (No Poverty) and SDG 8 (Decent Work and Economic Growth).^5 6^ Farmers are disproportionately impacted by climate change, which threatens crop yields, disrupts livelihoods and places them at the centre of SDG 13 (Climate Action).^7 8^ Farmers also work in one of the most hazardous sectors,^1^ where occupational risks directly impede the achievement of SDG 3 (Good Health and Well-Being).

### Health risks in agricultural work

Under SDG 3, targeted health outcomes include reducing premature mortality from non-communicable diseases (NCDs), reducing accidental deaths, combating communicable diseases and addressing mental health disorders, suicide and substance abuse.^4^ Agricultural workers face specific health challenges across these categories, including chronic exposure to agrochemicals, physical injuries, zoonotic diseases and psychosocial stressors.^9^,^14^ Farmers are at increased risk of NCDs, including respiratory, neurological and renal conditions, as well as cancers, often linked to chronic exposure to pesticides or particulate matter.^15 16^ Paradoxically, farmers also experience diet-related NCDs such as obesity, diabetes and cardiovascular diseases, often driven by food insecurity and limited access to diverse diets.^11 17^ Agriculture is one of the most hazardous sectors for occupational injuries globally. Farmers are facing high rates of machinery-related accidents, acute pesticide poisoning and repetitive strain injuries leading to high rates of musculoskeletal disorders.^1^ Farmers with close animal contact are at risk for zoonotic diseases such as brucellosis, bovine tuberculosis and cysticercosis.^18^ While advanced surveillance systems and animal health management can mitigate these risks, they persist in contexts with limited veterinary services and management systems.^14^ Additionally, the emergence of antibiotic-resistant pathogens in intensive livestock operations poses significant global concerns.^19^ The mental health of farmers is increasingly recognised as a critical concern, including chronic stress, anxiety, depression and suicide. Economic instability, climate-related pressures and social isolation exacerbate these challenges, particularly in rural areas with limited access to mental health services.^10^

These health risks are further exacerbated by climate change, which threatens livelihoods through declining crop yields, intensifies heat stress linked to kidney disease, increases the prevalence of vector-borne diseases like malaria and dengue, drives waterborne illnesses such as cholera, exacerbates respiratory issues due to poor air quality and heightens mental health challenges from economic instability to forced displacement, particularly in LMICs where adaptation resources are scarce.^7 8^

### Why context matters: comparing HICs and LMICs

The health risks faced by farmers differ significantly by variations in agricultural practices, safety regulations and healthcare infrastructure. For example, in industrialised farming, typical for HIC contexts, confined animal feeding operations contribute to respiratory issues from exposure to dust and mould, though modern ventilation systems may reduce these risks. Similarly, mechanisation reduces physical labour but increases the likelihood of machinery-related injuries such as tractor rollovers.^20^ Stricter pesticide regulations and access to personal protective equipment lower acute poisoning risks, yet long-term health effects such as cancer remain a concern.^21^

In contrast, in LMICs with a mix of industrialised and subsistence farming, burning crop residues, high exposure to dust in non-mechanised settings and inadequate ventilation in traditional storage facilities all increase the risk of respiratory diseases. Minimal mechanisation and inadequate safety measures can lead to higher rates of musculoskeletal disorders and tool-related injuries,^20^ while less stringent regulations and limited access to protective equipment heighten pesticide poisoning risks.^22^ Zoonotic diseases pose a greater threat due to closer human-animal contact and limited veterinary services.^14 18^ Many subsistence farmers face malnutrition and food insecurity,^17^ though obesity and cardiovascular diseases are rising.^23^ Furthermore, mental health issues are often under-recognised and undertreated due to limited healthcare access and cultural stigma.^24 25^

Understanding contextual variations in farmers’ health is crucial, given their pivotal role in advancing global food security and achieving the SDG agenda. However, it remains unclear where farmer health research is conducted and how health topics researched in farmer populations align with SDGs. Recent farmer strikes across the European Union, driven by dissatisfaction with agricultural policies and systemic neglect, highlight a growing disconnect between institutional priorities and farmers’ lived realities.^26^ Similar tensions have emerged in the USA and parts of the Global South,^27^ suggesting that the current research and policy agenda may not reflect the concerns most salient to farmers themselves. Systematically mapping global research trends in farmer health is essential to identify knowledge gaps, underserved regions and a potential mismatch between research and SDGs. This is particularly important in LMICs, where health risks are presumed to be highest, yet data remain limited.^28 29^

To address this gap, we conducted an artificial intelligence (AI)-assisted evidence map of research records to systematically screen and categorise the global literature on health research in farming populations from the adoption of the SDGs in 2015 through June 2024. Specifically, we aimed to:

1. Describe the geographical distribution of farmer health research activity, including differences by country income group (HIC vs. LMIC).
2. Characterise the topical focus of the literature by mapping records to major health outcome domains relevant to farming populations, including NCDs, communicable diseases, accidents and mental health.
3. Assess coverage and gaps relative to SDG 3 priorities by mapping topics with SDG 3 targets to identify disparities and opportunities for action.

## Methods

We conducted an evidence map of research records on health-related outcomes among farming populations across two main bibliographic health databases: Ovid/MEDLINE and Ovid/APA PsycInfo. The search covered records published from the adoption of the SDGs in 2015 to 19 June 2024.

The search strategy was designed to capture a broad range of health research focusing on farmer populations. We defined the farmer population using a single search block, targeting titles, abstracts and keywords, and incorporating relevant Medical Subject Headings (MeSH) terms. Animal studies indexed with MeSH terms were excluded to focus solely on human health outcomes. The complete search string and search limits for Ovid/MEDLINE are included in online supplemental materials S1.

This search resulted in 32 006 records. After deduplication, 31 503 unique records remained for review, of which we manually screened 8533. We screened titles and abstracts to identify records explicitly addressing health outcomes in farming populations. To manage screening at scale, we used SWIFT-Active Screener,^30^ a web-based collaborative systematic review application. This software employs active learning models to prioritise records for human review and provides integrated recall estimation based on labelled screening decisions.^31 32^ This means SWIFT-Active Screener influenced only the order in which records were screened; all inclusion/exclusion decisions and topic coding were made by human reviewers. The software was accessed via a time-limited institutional licence. To ensure the algorithm’s relevance and accuracy, bibliographic record information retrieved from the databases (titles, abstracts, keywords and associated metadata) of 90 seed articles representing a broad spectrum of health outcomes and study designs in farming populations were uploaded (online supplemental materials S2). These seed records were used to (1) conduct sensitivity checks to validate the retrieved results and (2) train the learning models to identify relevant studies, using the same title/abstract information available during screening. In addition, we conducted a bounded sensitivity screening of records published in 2025 as a methodological check on retrieval performance beyond the main search period; these records were not included in the formal evidence map, and details are provided in onlinesupplemental materials S3  S7.

Title/abstract screening was conducted using a single-screener approach (one reviewer per record) by a team of four reviewers. To support consistency, the team held regular meetings to refine decision rules and adjudicate borderline cases. In these instances, cases were reviewed collectively to ensure coding consistency and to iteratively refine the categorisation process. This approach reflects pragmatic methods often used in streamlined screening workflows, while recognising that it differs from dual independent screening used in many full systematic reviews.^30^

**Figure 1:**
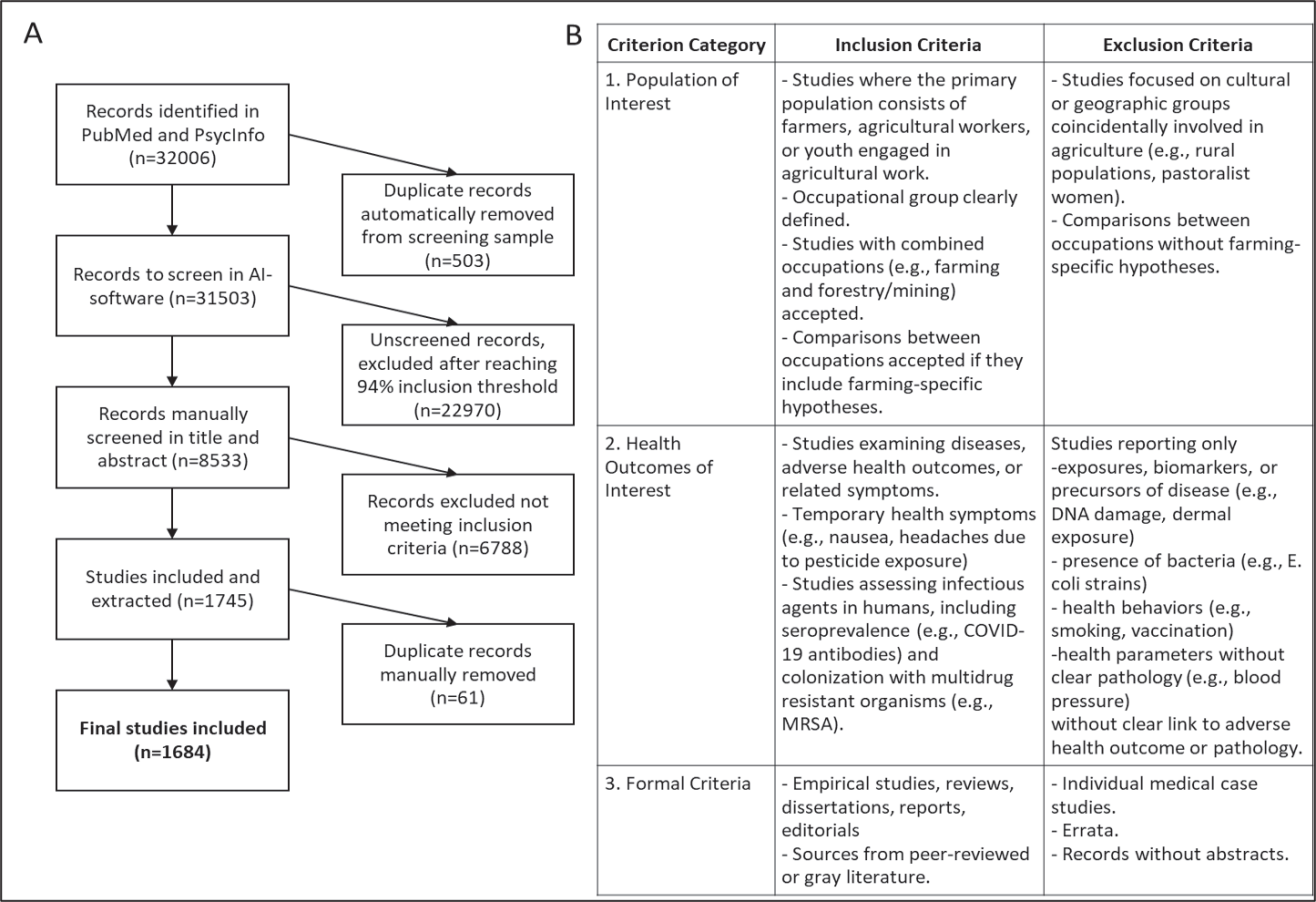
Study selection criteria. A) flow diagram of study selection, B) Inclusion and exclusion criteria. AI, artificial intelligence; *E. coli, Escherichia coli*; MRSA, methicillin-resistant *Staphylococcus aureus*.

Screening continued until the machine learning algorithm’s recall estimation indicated an estimated recall threshold of 0.94, consistent with the tool’s integrated recall estimation approach.^31 32^ This cut-off corresponds to SWIFT’s estimate that at least 94% of all relevant records in the retrieved set had been identified given the labelled decisions to that point. At that point, we had manually screened 8533 records. We selected this stopping threshold as a pragmatic balance between feasibility and completeness for an evidence map intended to describe broad patterns and gaps in the literature rather than to synthesise effects or appraise study quality. Accordingly, this evidence map describes research coverage and gaps and should not be interpreted as evidence of effectiveness or used to make causal inferences. The review process flow is depicted in figure 1, the Preferred Reporting Items for Systematic Review and Meta-Analysis extension for Scoping Reviews (PRISMA-ScR) checklist is included in online supplemental materials S4.

### Data extraction

Title and abstracts were used for data extraction. Studies reporting multiple outcomes were tagged for each health outcome. No information was extracted from full texts. Ambiguous cases or outcomes not fitting predefined categories were reviewed in coding meetings and reclassified if necessary. Because the unit of analysis was the publication record, we did not weight reviews differently from primary studies and did not adjust counts for overlapping underlying evidence across record types.

Health outcomes were classified into four main categories, adapted from the Global Burden of Disease (GBD) level 1 classification^33^: (1) Communicable diseases, (2) Non-communicable diseases, (3) Accidents and injuries and (4) Mental health issues. We used the GBD level 1 structure as a pragmatic, widely used framework to support consistent categorisation across a diverse and multidisciplinary literature and to facilitate mapping of topics to SDG 3 priorities. We then incorporated subcategories based on relevant SDG 3 indicators,^4^ where applicable. Additional categories, such as pesticide poisoning and musculoskeletal disorders, were introduced based on the literature and ongoing coding results, with particular focus on outcomes pertinent to farmers. For pesticide-related poisoning, we coded records describing pesticide ingestion as intentional self-harm/suicide under *mental health*, and records describing unintentional pesticide poisoning or occupational exposure under *accidents and injuries*, based on the framing and explicit wording in the title/abstract. Through iterative refinement, we continuously adjusted category definitions and introduced new categories when clusters of studies on specific outcomes emerged. This process aimed to minimise the ‘other health outcomes’ category while avoiding excessive subcategorisation, ensuring the focus remained on capturing major trends in health outcomes. Online supplemental materials S5 provides an overview of health outcome categories and their definitions.

Geographical location was extracted as reported in the title and abstract and reflects the location where the study was conducted (ie, the geographical location of the research), not the participants’ place of origin. We did not reclassify cross-border or non-administrative populations beyond what was stated in the record. Country names and regions were categorised into HICs and LMICs based on the World Bank’s 2023 income group classification.^34^ For studies that mentioned specific cohorts without providing a geographical location, such as the Agricultural Health Study (AHS, USA) or Agriculture and Cancer cohort (AGRICAN, France), the geographical location was completed using additional cohort-specific information. Studies with a regional focus (eg, Nordic countries, sub-Saharan Africa; n=13) were excluded from map visualisations but were manually assigned to the most appropriate HIC or LMIC category based on their regional context. Studies reporting results from multiple specific countries (n=25) were mapped individually for each country in the map analyses to reflect the distribution more accurately.

### Data analysis

Bibliographic information and screening results for included and excluded studies were exported from the SWIFT-Active Screener^32^ as CSV and Excel files, each linked with a unique identifier. The data were then manually prepared for analysis. Specifically, we coded country information into International Organization for Standardization 3166–1 alpha-3 (ISO3) codes, streamlined ‘other’ codes and merged the country-level data with World Bank data on HIC and LMIC income groups.^34^ Following data integration, we conducted minor cleaning and recoding of multiple outcome variables in Stata.^35^ We also identified and removed 61 duplicate records that had been overlooked by the software’s duplicate detection system. These duplicates were retained in the screening pool initially to prevent confusion in the algorithm’s identification process. Details and citations for all included records are provided in online supplemental dataset S6 (csv).

Data visualisation was performed using the R package ggplot2^36^ and the rnaturalearth package.^37^ This allowed us to generate world maps illustrating the global distribution of studies. To visualise the agricultural workforce, we incorporated ILO modelling data^38^ into the world maps, providing context for the health research trends observed.

## Results

We conducted an AI-assisted evidence map of research records of published literature on health-related outcomes among farming populations across two main health databases and identified 32 006 possibly relevant records. We manually screened 8533 records when the cut-off of 94% of eligibility was reached in the systematic review software^32^ and included a total of 1684 studies in our analyses. See figure 1 for a flowchart of study selection (1A) and detailed inclusion and exclusion criteria (1B). We extracted country information from 1418 records and regional information from 14 studies. 116 studies (7%) had a global focus and 136 studies (8%) did not provide sufficient geographical information in the abstract and were thus excluded from geographical analyses.

**Figure 2:**
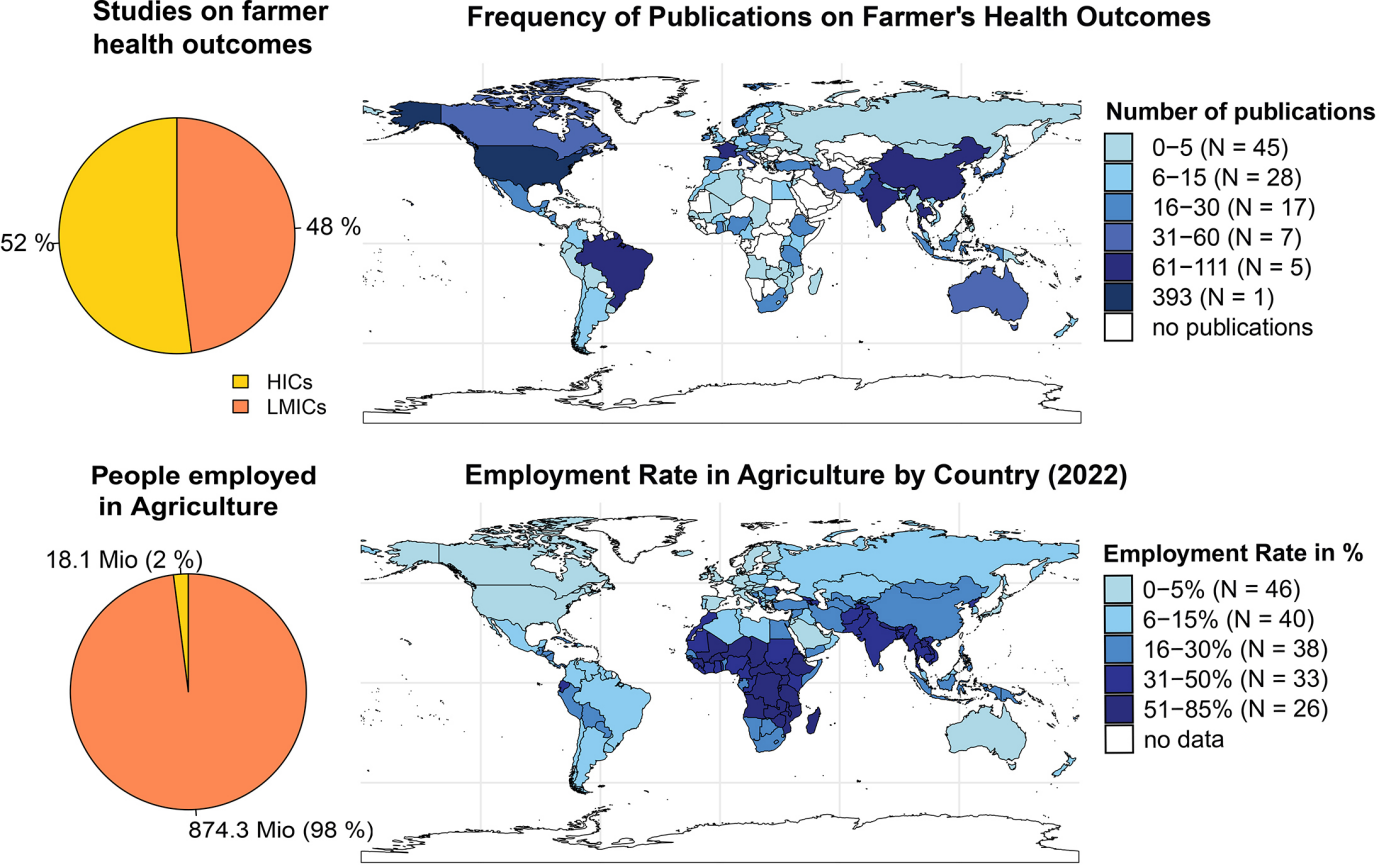
Research output by country in relation to population working in agriculture and in LMICs versus HICs. HICs, high-income countries; LMICs, low- and middle-income countries.

We extracted 1469 geographical locations (25 studies were conducted in multiple countries) with nearly equal contributions from HICs (765 studies, 52%) and LMICs (704 studies, 48%), meaning that although LMICs make up 98% of the global agricultural workforce (874 million people compared with 18.1 million in HICs), research output is still highly concentrated in HICs, with over 50% of the research representing just 2% of the affected population (see figure 2 research output by country).

Most studies were published using US samples (n=341, HIC), with more than twice the contributions than the next two countries, China (n=93, LMIC) and India (n=66, LMIC), together. The second and third rank of contributions from HIC countries came from France (n=54) and South Korea (n=50).

**Figure 3:**
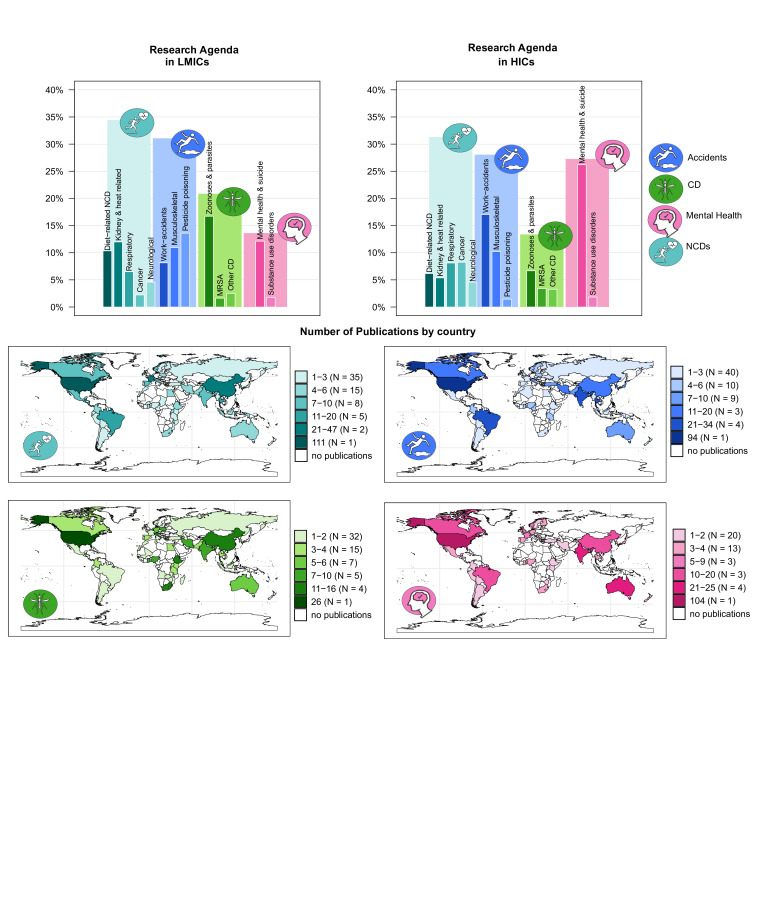
Research topics in LMICs versus HICs). CD, communicable disease; HICs, high-income countries; LMICs, low- and middle-income countries; MRSA, methicillin-resistant *Staphylococcus aureus*; NCD, non-communicable disease.

The most-studied health outcomes were NCDs, which accounted for 33% of studies across both income groups (see figure 3 global research topics by country). However, on the subcategory level, studies from LMICs focused more frequently on diet-related NCDs (10% vs 6% in HIC samples) and kidney and heat-related illnesses (11% vs 5% in HIC samples), while studies from HICs focused more on respiratory conditions (8% vs 6% in LMICs) and cancer (8% vs 2% in LMICs; see table 1). Accidents and injuries represented 29% of studies in both groups, though work-related injuries were more frequently investigated in HICs (17% vs 8% in LMICs), and pesticide poisoning was almost exclusively researched in LMICs (13% vs 1% in HICs).

**Table 1:** Research topics by frequency of studies, overall and by HIC/LMIC country

	All studies*		HIC**		LMIC**	
	Overall number of studies	Percentage of Studies	Number of samples by country	Percentage of Studies	Number of samples by country	Percentage of Studies
All categories	1684	100	769	100	704	100
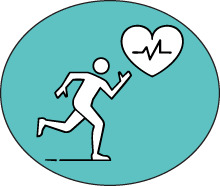 **Non-communicable diseases**	558	33	238	31	234	33
Diet-related NCD (cardiovascular, diabetes, obesity)	123	7	46	6	70	10
Kidney and heat related illness	138	8	41	5	81	11
Respiratory illness	151	9	62	8	44	6
Cancer	85	5	64	8	15	2
Neurological conditions	85	5	34	4	32	5
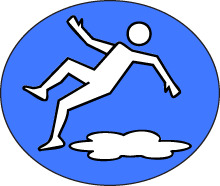 **Accidents and injuries**	481	29	213	28	211	30
Work-accidents	199	12	130	17	55	8
Musculoskeletal problems	186	11	77	10	75	11
Pesticide poisoning	112	7	11	1	92	13
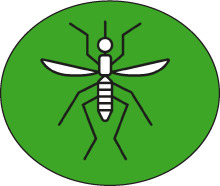 **Communicable diseases**	270	16	101	13	142	20
Zoonoses & parasites	177	11	50	7	114	16
MRSA	47	3	26	3	11	2
Other CD	46	3	25	3	17	2
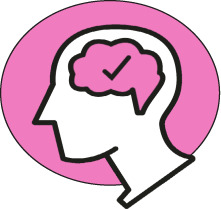 **Mental health**	337	20	210	27	92	13
Mental health and suicide	317	19	202	26	82	12
Substance use disorders	28	2	14	2	12	2
General health and well-being	97	6	35	5	57	8
Other health outcomes	108	6	42	5	54	8

*All included studies, including 136 studies with missing geographical information and 116 studies with a global perspective; **Studies with any country or regional info n=1432. Studies with samples from multiple countries (n=25) were counted for each country/sample. Total amounts to more than 100% since studies with multiple outcomes were counted for each outcome.

CD, communicable disease; HICs, high-income countries; LMICs, low- and middle-income countries; MRSA, methicillin-resistant *Staphylococcus aureus*; NCD, non-communicable disease.

Communicable disease research (16%) was more prominent in LMICs (20% vs 13% in HICs), particularly on zoonoses and parasitic infections (16% vs 7% in HICs). Mental health, including suicide, made up 20% of studies, with a stronger focus in HICs (27% vs 12% in LMICs). Substance use disorders were rarely studied, representing only 2% of all research, with limited coverage in both settings.

**Figure 4:**
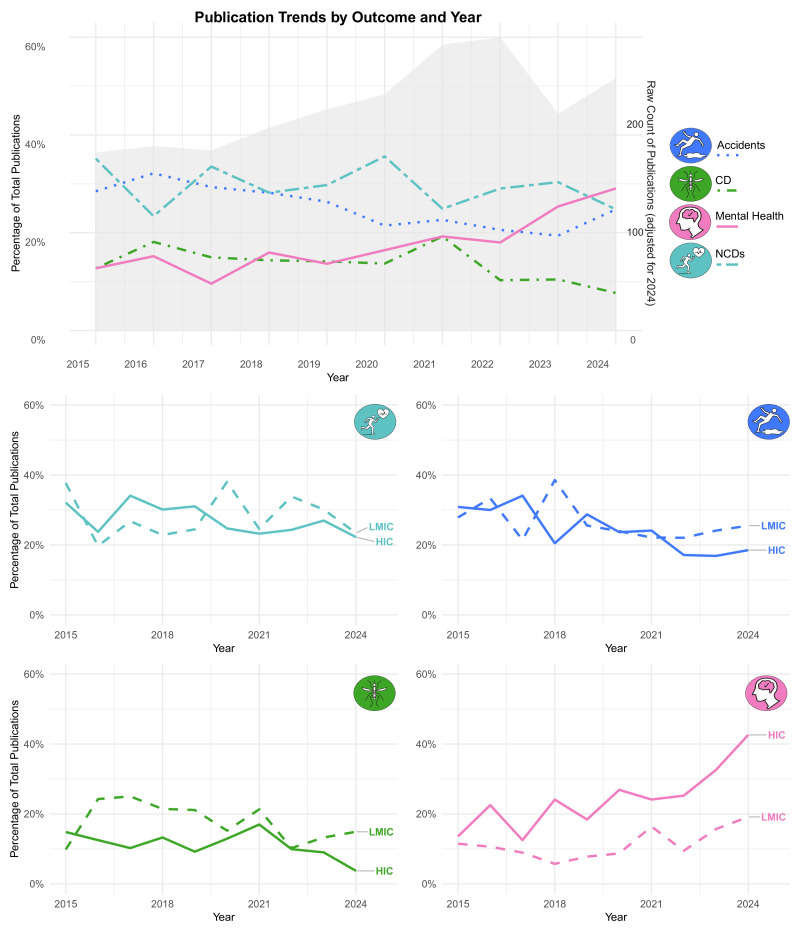
Percentage of publications by outcome and year, as well as by high income and low-income contexts. HICs, high-income countries; LMICs, low- and middle-income countries; NCD, non-communicable disease.

Between 2015 and 2024, mental health emerged as a growing focus in the included studies (see figure 4). The proportion of publications addressing mental health steadily increased, particularly from 2021 onwards, rising from under 15% to nearly 30% of all publications by 2024. This upward trend was observed in both HICs and LMICs, with LMICs showing a particularly sharp rise over the last 3 years. In contrast, publications on CDs declined over time, most markedly in HICs, where interest has waned considerably since 2021. While LMICs maintained relatively consistent levels of attention to communicable diseases through 2022, a similar downward trajectory was seen more recently. NCDs and accidents showed no clear directional trend, with publications on both topics remaining relatively stable. However, there was a slight downward trend for publications on accidents, and some periodic peaks in publications on NCDs in 2020 and 2022/2023, particularly in LMICs. Together, these findings suggest a shifting research agenda, with increasing emphasis on mental health in farming populations, potentially reflecting evolving policy priorities and global health discourse.

## Discussion

This AI-assisted evidence map of research records (32 006 retrieved; 1684 included) provides a global overview of farmer health research activity from 2015 to 2024 and highlights substantial imbalances in the evidence base.

### Health research in farmer populations comparing HIC and LMIC contexts

Although 98% of the agricultural workforce resides in LMICs, over half of the research originated in HICs, with the USA alone accounting for 24%. Across settings, NCDs were the most frequently studied topic (33% of studies). However, notable divergences in research priorities between income contexts emerged. Pesticide poisoning and communicable diseases dominated LMIC-focused studies, while mental health research was more common in HICs. Since 2021, publications on mental health have sharply increased, suggesting growing awareness of psychosocial stressors and its impact among farmers. Although several themes reflect SDG 3 targets, the mapped literature suggests that key determinants of farm workers’ health, such as occupational protections, labour conditions and access to services, remain unevenly studied, especially in LMIC settings.

NCDs dominate the literature, particularly in HICs, reflecting both the global burden of diet-related conditions among farmers and a research agenda shaped by settings where these diseases drive morbidity. This focus is reinforced by large-scale cohort studies like those in the international Agricultural Cohort Consortium (AGRICOH),^39^,^42^ AHS^43^,^46^ and AGRICAN,^47^,^49^ which enable robust investigation of other chronic health outcomes, such as cancer. Musculoskeletal disorders and accidental injuries were also key topics across settings. Given farming’s physical demands, this is unsurprising. The focus on work accidents in HICs likely reflects stronger occupational reporting systems and robust occupational health surveillance data, along with a high grade of mechanisation.^50 51^ This suggests the evidence base is shaped not only by burden, but by surveillance capacity, funding and regulatory infrastructures, which may bias what becomes visible in the literature. This disconnect between research trends and global policy priorities raises important questions about whose health is measured and prioritised, and suggests that the research agenda is often shaped by the capacity and infrastructure of HICs rather than by global need.

Mental health and suicide represented 20% of studies from HICs and 13% from LMICs. Despite a growing interest in mental health research globally, public health responses remain limited and lag far behind strategic planning for communicable diseases and other NCDs.^52^ Illustrative, a significant share (31%) of mental health studies drew on farmer samples from the USA, a country where institutional surveillance and policy might lend more support for this research topic. In contrast, despite the WHO’s inclusion of mental health in the global development agenda, surveillance and formal mental health policies are sparse in LMICs.^53^ Differences in environmental exposure and regulation are also apparent. Studies on pesticide poisoning were concentrated in LMICs, where informal employment, limited worker protections and weak safety enforcement elevate risk.^54 55^ Similarly, renal disease research was disproportionately studied in LMIC contexts, where rising temperatures and labour-intensive agriculture may pose direct threats to kidney health.^56^ Communicable disease research also diverged by context. While LMICs focused on zoonotic infections, HICs research emphasised antimicrobial resistance (eg, methicillin-resistant *Staphylococcus aureus*)^57^ and tick-borne infections, particularly in the northern hemisphere—where occupational exposure for outdoor workers is a concern.^58^

NCDs and injury-related research remained relatively stable over the study period, suggesting persistent structural and occupational risks. In contrast, research on communicable diseases declined sharply post-2021, particularly in HICs, despite ongoing concerns about zoonoses and antimicrobial resistance in farming contexts.^18 29^ Mental health research has gained prominence since 2021, with the proportion of publications nearly doubling between 2021 and 2024 in both income contexts, suggesting a critical shift in research attention. This rise may be linked to the lingering psychosocial effects of the COVID-19 pandemic, coupled with massive global policy efforts,^59^ and growing awareness of increasing stressors posed by climate change and economic precarity in rural communities.^10^

### Alignment of conducted research with SDG 3 targets

SDG 3 aims to ‘ensure healthy lives and promote well-being for all at all ages’, emphasising NCDs, mental health, injuries and infectious diseases. While many of these areas intersect with the health needs of farming populations, our findings reveal both important alignments and critical omissions: Communicable diseases, such as neglected tropical diseases and zoonoses, are a priority under SDG 3, and were widely studied, particularly in LMICs.^60 61^ However, other major vector-borne diseases like malaria—an explicit SDG 3 target—were under-represented. This may reflect the framing of malaria as a general public health issue rather than an occupational hazard, despite farmers’ high exposure.^62^ NCDs such as respiratory disease,^40^ cardiovascular^63^ and cancer^42^ conditions were commonly studied, particularly in HICs where major long-term agricultural cohorts exist (eg, within the AGRICOH consortium), aligning with SDG 3 priorities. Diet-related conditions like hypertension or obesity also featured prominently.^23^ However, diabetes, despite being a leading global cause of mortality^64^ and an SDG 3 priority, was strikingly under-represented. This gap may stem from limited occupational framing or difficulty isolating agricultural-specific risk factors. Similarly, road traffic injuries, which is an SDG 3 priority, were rarely examined in farming populations, likely because they are not framed as occupational hazards even though rural transport and machinery use make them relevant. Finally, mental health is included under SDG 3, and its rising prominence in the literature—particularly from HICs—suggests increasing recognition of the psychosocial burdens in agriculture.^10 65^ Still, mental health remains weakly integrated into national health strategies, especially in LMICs.^53^ However, while recognised in SDG 3, substance use was addressed in fewer than 30 studies, a striking gap given rising alcohol and opioid use in rural and farming populations in many countries.^66 67^

Several high-burden occupational health outcomes lie outside SDG 3’s scope.

Musculoskeletal disorders and occupational injuries are highly prevalent and disabling in farming populations, yet they are not part of the SDG 3 targets, despite their major contribution to years lived with disability globally.^68^ In farming populations, these outcomes are closely linked to working conditions and the physical demands of agricultural labour; their omission from SDG 3 monitoring limits accountability for reducing occupation-related harm and tracking progress toward farm workers’ rights to health; their omission from SDG 3 monitoring limits accountability for reducing occupation-related harm and tracking progress toward farm workers’ rights to health. More broadly, this omission reflects a failure to capture the lived realities of millions of farmers.

Similarly, renal dysfunction, heat-related illness and chronic kidney disease of unknown origin appeared as highly important topics, particularly for outdoor agricultural workers in LMICs where high heat exposure and manual labour exacerbate risk.^56 69^ These climate-sensitive conditions are not captured in SDG 3 monitoring frameworks, revealing the limited responsiveness of global health priorities to rapidly evolving environmental risks. Indeed, improving outcomes under SDG 3 also supports progress on other SDGs, such as SDG 8 on Decent Work and Economic Growth.^70^ These goals are closely interlinked, as decent work conditions—such as fair wages, job security and safe workplaces—contribute significantly to both physical and mental well-being.^71^ Explicit inclusion of agricultural worker–relevant occupational indicators would strengthen SDG-era monitoring and support policy action on preventable harm in farming populations.

### Implications for policy, research and post-2030 agendas

As agricultural systems undergo rapid transformation to meet the projected doubling of food production by 2050, protecting the health of the farming workforce is not optional, it is essential. Looking ahead to the post-2030 era, closing the gaps identified in this evidence map will require both improved measurement and stronger integration of occupational health into sustainability agendas. Practical priorities include strengthening occupational health surveillance and reporting, integrating occupational health within health systems and climate adaptation planning and investing in research capacity and data generation in LMIC agricultural settings. Integrative frameworks such as One Health,^72^ which recognises the interdependence of human, animal and environmental health, and the Exposome Concept,^73^ which captures the cumulative impact of lifetime exposures, offer powerful examples for embedding health into agricultural transformation. Simultaneously, the growing global focus on mental health must expand to include LMICs. As climate change, digitalisation, economic instability, environmental degradation and social marginalisation intensify pressures on rural populations, investing in mental health research and services will be essential. As the world prepares for the post-2030 era, the UN Pact for the Future^74^ could offer a timely opportunity to embed occupational health, mental well-being and environmental justice more centrally into a global health strategy for a resilient agricultural workforce.

### Study strengths and limitations

This review had certain limitations: (1) we included only studies explicitly addressing health in occupational agricultural contexts, potentially excluding broader rural health research, particularly in LMICs where informal work is common. As a result, part-time, seasonal or unpaid agricultural labour, often performed by women, children or older adults, may be under-represented.^75^ This may be particularly relevant in settings where farming is subsistence-based or one of multiple occupations, and where informal agricultural work is common. (2) Studies comparing farmers to other professions were excluded unless farmers were a distinct subgroup, which may have limited broader trend analysis. (3) Reliance on indexed and published literature introduces potential publication and language biases. (4) Screening was supported by SWIFT-Active Screener, which uses active learning to prioritise records for review and integrated recall estimation to support stopping decisions.^31 32^ The tool did not replace human judgement: it influenced only the order in which records were screened, while all inclusion decisions and topic classifications were made manually based on titles/abstracts. Nonetheless, active-learning prioritisation combined with abstract-only screening may lead to residual under-ascertainment or misclassification, particularly for niche topics or poorly described abstracts. While we consider the estimated recall threshold of 0.94 of the tool appropriate for describing broad trends in an evidence map, we acknowledge that it does not guarantee complete capture of all relevant records. We also acknowledge potential concerns related to the vendor’s reported financial links to British American Tobacco; however, the software could not influence results beyond screening order, since all coding decisions were made by the research team. Finally, because we mapped research records based on titles/abstracts without full-text extraction or critical appraisal, the findings describe the distribution of research activity and should not be interpreted as evidence of intervention effectiveness or causal relationships. In addition, research priorities may shift over time; therefore, newer and emerging themes published after our search date may not yet be captured in this evidence map. Despite these limitations, this evidence map offers a large-scale overview of geographical and topical patterns of health research literature during 2015–2024 within the world’s largest occupational sector.

### Conclusions

This AI-assisted evidence map provides critical insight into global research patterns on farmers’ health, revealing a misalignment between the health research conducted on farming populations, the burden of disease and global policy priorities. While several SDG 3-aligned issues, such as mental health, cardiovascular disease and infectious disease, are well represented, other pressing health issues receive comparatively little attention. Musculoskeletal conditions, injuries and heat-related illness are high-burden occupational risks in farming, yet they are incompletely captured in SDG 3 targets and indicators.

Another key finding is the geographical disconnect between where agricultural workers live and where research is produced: the evidence base disproportionately reflects HIC settings, while LMIC evidence remains comparatively sparse and often focused on acute risks like pesticide exposure or zoonotic infections. This leaves broader structural determinants, such as working conditions, occupational protections and access to services, underexplored in the literature. In LMICs, farming is often a central livelihood for large portions of the population, making this knowledge gap especially problematic for public health planning.

Overall, these findings highlight the need for a more balanced and context-sensitive research agenda on farmers’ health, particularly in under-represented LMIC settings and for neglected occupational health outcomes. Addressing these gaps will be important for informing more equitable public health and policy responses.

## Supplementary material

10.1136/bmjopen-2025-110537 online supplemental file 1

10.1136/bmjopen-2025-110537 online supplemental file 2

10.1136/bmjopen-2025-110537 online supplemental file 3

10.1136/bmjopen-2025-110537 online supplemental file 4

10.1136/bmjopen-2025-110537 online supplemental file 5

10.1136/bmjopen-2025-110537 online supplemental file 6

10.1136/bmjopen-2025-110537 online supplemental file 7

## Data Availability

Data are available upon reasonable request.
