# Supplementary material for: Unequal harvests: AI-assisted evidence map of trends and gaps in global farmer health research along SDG 3 priorities
Source: BMJ Open. 2026 Jun 1;16(6):e110537. doi: 10.1136/bmjopen-2025-110537 (PMC13239455; doi:10.1136/bmjopen-2025-110537)
Supplement: online supplemental file 1 [file bmjopen-16-6-s001.pdf]

Supplementary Materials S1.

Complete search string for Ovid/Medline

1. (exp farmers/ or (farmer* or "*grower*" or ranch* or harvester or picker or breeder or winemaker or pastoral* or herder or planter or ((farm* or agricultur* or horticultur* or food) adj3 (work* or producer*))).ti,ab,kw.) NOT (exp animals/ NOT exp humans/)

2. Limit 1 to yr="2015 -Current"

Limits applied: yr=2015–Current (via Ovid Limits interface)

Search run date: Search executed on 19 June 2024; ‘Current’ corresponds to records indexed up to that date.
