# Supplementary material for: Unequal harvests: AI-assisted evidence map of trends and gaps in global farmer health research along SDG 3 priorities
Source: BMJ Open. 2026 Jun 1;16(6):e110537. doi: 10.1136/bmjopen-2025-110537 (PMC13239455; doi:10.1136/bmjopen-2025-110537)
Supplement: online supplemental file 2 [file bmjopen-16-6-s002.pdf]

Supplementary Materials S2. List of seed articles for AI training

Authors	Year	Title	Topic
Acharya et al.	2022	Acharya, A., & Panda, A. (2022). Clinical epidemiology and predictors of outcome in chlorpyrifos poisoning in farming and allied agricultural workers in East Godavari, Andhra Pradesh. Indian journal of occupational and environmental medicine, 26(2), 116-121.	Poisoning
Adandom et al.	2023	Adandom, H. C., Ofori-Dei, S. M., & Hallstrom, L. K. (2023). Health and well-being of Hutterite farmers in Alberta: Results from the Sustainable Farm Families Alberta program. Canadian Journal of Rural Medicine, 28(3), 123-130.	Other health outcome
Adibell et al.	2024	Adibellİ, D., & Sümen, A. (2024). Non-fatal agricultural injuries and first aid self-efficacy among greenhouse workers in Turkey. Journal of agromedicine, 29(1), 1-9	Accident
Afshari et al.	2018	Afshari, M., Poorolajal, J., Assari, M. J., Rezapur-Shahkolai, F., & Karimi-Shahanjarini, A. (2018). Acute pesticide poisoning and related factors among farmers in rural Western Iran. Toxicology and industrial health, 34(11), 764-777.	Poisoning
Akbar et al.	2023	Akbar, K. A., Try, P., Viwattanakulvanid, P., & Kallawicha, K. (2023). Work-related musculoskeletal disorders among farmers in the Southeast Asia region: A systematic review. Safety and health at work, 14(3), 243-249.	Musculoskeletal
Ali er al.	2021	Ali, Danish, Kaiser, Muhammad Adeel, Khokhar, Muhammad Imran et al.. Fodder cutter (Tokka) injuries: A preventable morbidity.. JPMA. The Journal of the Pakistan Medical Association, 2021, 71: 1022-1024	Accident
Amoatey et al.	2020	Amoatey, P., Al-Mayahi, A., Omidvarborna, H., Baawain, M. S., & Sulaiman, H. (2020). Occupational exposure to pesticides and associated health effects among greenhouse farm workers. Environmental Science and Pollution Research, 27, 22251-22270.	Poisoning
Anakwue	2019	Anakwue, R. (2019). Cardiotoxicity of pesticides: are Africans at risk?. Cardiovascular toxicology, 19(2), 95-104.	Poisoning
Andreotti	2018	Andreotti, G., Koutros, S., Hofmann, J. N., Sandler, D. P., Lubin, J. H., Lynch, C. F., Lerro, C. C., De Roos, A. J., Parks, C. G., Alavanja, M. C., Silverman, D. T., & Beane Freeman, L. E. (2018). Glyphosate Use and Cancer Incidence in the Agricultural Health Study. Journal of the National Cancer Institute, 110(5), 509–516. Ovid MEDLINE(R) <2018>. https://doi.org/10.1093/jnci/djx233	Cancer
Andreotti	2020	Andreotti, G., Beane Freeman, L. E., Shearer, J. J., Lerro, C. C., Koutros, S., Parks, C. G., ... & Hofmann, J. N. (2020). Occupational pesticide use and risk of renal cell carcinoma in the agricultural health study. Environmental health perspectives, 128(6), 067011.	Cancer
Arcury et al.	2016	Arcury, T. A., Talton, J. W., Summers, P., Chen, H., Laurienti, P. J., & Quandt, S. A. (2016). Alcohol consumption and risk for dependence among male Latino migrant farmworkers compared to Latino nonfarmworkers in North Carolina. Alcoholism: clinical and experimental research, 40(2), 377-384.	Substance use
Armendáriz-Arnez et al.	2023	Armendáriz-Arnez, C., Tamayo-Ortiz, M., Mora-Ardila, F., Rodríguez-Barrena, M. E., Barros-Sierra, D., Castillo, F., ... & Mora, A. M. (2023). Prevalence of SARS-CoV-2 infection and impact of the COVID-19 pandemic in avocado farmworkers from Mexico. Frontiers in Public Health, 11, 1252530.	Any Communicable disease
Arphom et al.	2022	Arphorn, S., Ishimaru, T., Lertvarayut, T., Kiatkitroj, K., Theppitak, C., Manothum, A., & Hara, K. (2022). Risk Factors for Occupational Falls among Middle-aged and Elderly Farm Workers in Nan Province, Thailand. Journal of Agromedicine, 27(4), 402–408. https://doi.org/10.1080/1059924X.2022.2040071	Accident

Arphom et al.	2021	Arphorn, S., Lertvarayut, T., Kiatkitroj, K., Theppitak, C., Manothum, A., Hara, K., & Ishimaru, T. (2021). Association between physical capacity and occupational falls among middle-aged and older farmers in Thailand: Using the self-check risk assessment tool in Japan. Journal of occupational health, 63(1), e12287.	Accident
Basher et al.	2015	Basher, A., Nath, P., Siddique, Z. S., Rahman, M. H., Rubel, M. A., Sayed, M. S., ... & Bhuiyan, M. R. (2015). Musculoskeletal disorder (MSD) among agricultural workers. Mymensingh medical journal: MMJ, 24(1), 1-8.	Musculoskeletal
Boggess et al.	2016	Boggess, B., & Bogue, H. O. (2016). The health of US agricultural worker families: A descriptive study of over 790,000 migratory and seasonal agricultural workers and dependents. Journal of health care for the poor and underserved, 27(2), 778-792	Multi-topic
Boulanger et al.	2018	Boulanger, M., Tual, S., Lemarchand, C., Guizard, A. V., Delafosse, P., Marcotullio, E., ... & Lebailly, P. (2018). Lung cancer risk and occupational exposures in crop farming: results from the AGRIculture and CANcer (AGRICAN) cohort. Occupational and Environmental Medicine, 75(11), 776-785.	Cancer
Boulanger et al.	2017	Boulanger, M., Tual, S., Lemarchand, C., Guizard, A.-V., Velten, M., Marcotullio, E., Baldi, I., Clin, B., & Lebailly, P. (2017). Agricultural exposure and risk of bladder cancer in the AGRIculture and CANcer cohort. International Archives of Occupational and Environmental Health, 90(2), 169–178. https://doi.org/10.1007/s00420-016-1182-y	Cancer
Brew et al.	2016	Brew, Bronwyn, Inder, Kerry, Allen, Joanne et al.. The health and wellbeing of Australian farmers: a longitudinal cohort study.. BMC public health, 2016, 16	Multi-topic
Cabréra et al.	2022	Cabréra, L., Auguste, A., Michineau, L., Joachim, C., Deloumeaux, J., & Luce, D. (2022). Lung cancer in the French West Indies: role of sugarcane work and other occupational exposures. International Journal of Environmental Research and Public Health, 19(20), 13444	Cancer
Caffaro et al.	2017	Caffaro, F., Micheletti Cremasco, M., Roccato, M., & Cavallo, E. (2017). It does not occur by chance: A mediation model of the influence of workers' characteristics, work environment factors, and near misses on agricultural machinery-related accidents. International journal of occupational and environmental health, 23(1), 52-59.	Accident
Chaikitmongkol et al.	2015	Chaikitmongkol, V., Leeungurasatien, T., & Sengupta, S. (2015). Work-related eye injuries: important occupational health problem in Northern Thailand. Asia-Pacific journal of ophthalmology, 4(3), 155-160.	Accident
Chang et al.	2023	Chang, C. J., & Yang, H. Y. (2023). Chronic kidney disease among agricultural workers in Taiwan: a Nationwide Population-Based Study. Kidney International Reports, 8(12), 2677-2689.	Renal
Cheney et al.	2022	Cheney, A. M., Barrera, T., Rodriguez, K., & Jaramillo López, A. M. (2022). The Intersection of Workplace and Environmental Exposure on Health in Latinx Farm Working Communities in Rural Inland Southern California. International Journal of Environmental Research and Public Health, 19(19), 12940.	Other Non-communicable disease
Chicas et al.	2022	Chicas, R., Xiuhtecutli, N., Houser, M., Glastra, S., Elon, L., Sands, J. M., ... & Hertzberg, V. (2022). COVID-19 and agricultural workers: a descriptive study. Journal of immigrant and minority health, 1-7.	Any Communicable disease
Clay et al.	2017	Clay, L., Milosavljevic, S., Koehncke, N., Dosman, J., & Trask, C. (2017). Trends of ATV use and associated injury on Saskatchewan farms. Journal of Occupational and Environmental Hygiene, 14(11), 853–862. https://doi.org/10.1080/15459624.2017.1339049	Accident
Cremonini et al.	2023	Cremonini, A. C. P., Ferreira, J. R. S., Martins, C. A., Do Prado, C. B., Petarli, G. B., Cattafesta, M., & Salaroli, L. B. (2023). Metabolic Syndrome and Associated Factors in Farmers in Southeastern Brazil: A Cross-Sectional Study. International Journal of Environmental Research and Public Health, 20(14), 6328.	Other Non-communicable disease
Daghagh et al.	2019	Daghagh Yazd, Sahar, Wheeler, Sarah Ann, Zuo, Alec. Key Risk Factors Affecting Farmers' Mental Health: A Systematic Review.. International journal of environmental research and public health, 2019, 16	Mental Health
De Oliveira et al.	2021	Santos, Emelynne Gabrielly de Oliveira, Queiroz, Paulo Roberto, Nunes, Aryelly Dayane da Silva et al.. Factors Associated with Suicidal Behavior in Farmers: A Systematic Review.. International journal of environmental research and public health, 2021, 18	Mental Health

de-Assis et al.	2020	de-Assis, M. P., Barcella, R. C., Padilha, J. C., Pohl, H. H., & Krug, S. B. F. (2020). Health problems in agricultural workers occupationally exposed to pesticides. Revista Brasileira de Medicina do Trabalho, 18(3), 352.	Poisoning
DeWit et al	2015	DeWit, Y., Pickett, W., Lawson, J., Dosman, J., & for the Saskatchewan Farm Injury Cohort Team. (2015). Farm activities and agricultural injuries in youth and young adult workers. Journal of agromedicine, 20(3), 318-326.	Accident
Di Gennaro et al.	2021	Di Gennaro, F., Lattanzio, R., Falanga, C., Negri, S., Papagni, R., Novara, R., ... & Saracino, A. (2021). Low-wage agricultural migrant workers in Apulian ghettos, Italy: general health conditions assessment and HIV screening. Tropical Medicine and Infectious Disease, 6(4), 184.	Multi-topic
Emiral et al.	2021	Emiral, G. O., Onsuz, M. F., Ozay, O., Isikli, B., & Metintas, S. (2021). Cardiovascular Disease Risk Factors among Migrant Seasonal Agricultural Workers: Comparison with Local Residents. Iranian Journal of Public Health, 50(4), 747	Cardiovascular
Farnham et al.	2021	Farnham, A., Fuhrimann, S., Staudacher, P., Quirós-Lépiz, M., Hyland, C., Winkler, M. S., & Mora, A. M. (2021). Long-term neurological and psychological distress symptoms among smallholder farmers in Costa Rica with a history of acute pesticide poisoning. International journal of environmental research and public health, 18(17), 9021.	Neurological
Finhler et al.	2023	Finhler, Suelen, Marchesan, Gabriela Putton, Corona, Cristian Ferreira et al.. Influence of pesticide exposure on farmers' cognition: A systematic review.. Journal of neurosciences in rural practice, 2023, 14: 574-581	Neurological
Fuhriman et al.	2019	Fuhrimann, Samuel, Winkler, Mirko S, Staudacher, Philipp et al.. Exposure to Pesticides and Health Effects on Farm Owners and Workers From Conventional and Organic Agricultural Farms in Costa Rica: Protocol for a Cross-Sectional Study.. JMIR research protocols, 2019, 8	Multi-topic
Fuhrimann et al.	2021	Fuhrimann, S., Farnham, A., Staudacher, P., Atuhaire, A., Manfioletti, T., Niwagaba, C. B., ... & Mora, A. M. (2021). Exposure to multiple pesticides and neurobehavioral outcomes among smallholder farmers in Uganda. Environment international, 152, 106477.	Neurological
Fuhrimann et al.	2023	Fuhrimann, S., Mueller, W., Atuhaire, A., Ohlander, J., Mubeezi, R., Povey, A., ... & Kromhout, H. (2023). Self-reported and urinary biomarker-based measures of exposure to glyphosate and mancozeb and sleep problems among smallholder farmers in Uganda. Environment International, 182, 108277.	Other health outcome
Gesesew et al.	2016	Gesesew, Hailay Abrha, Woldemichael, Kifle, Massa, Desalegn et al.. Farmers Knowledge, Attitudes, Practices and Health Problems Associated with Pesticide Use in Rural Irrigation Villages, Southwest Ethiopia.. PloS one, 2016, 11	Poisoning
Gomaa et al.	2020	Gomaa, M. R., El Rifay, A. S., Zeid, D. A., Elabd, M. A., Elabd, E., Kandeil, A., ... & Kayali, G. (2020). Incidence and seroprevalence of avian influenza in a cohort of backyard poultry growers, Egypt, August 2015–March 2019. Emerging infectious diseases, 26(9), 2129.	Any Communicable disease
Guillien et al-	2016	Guillien, A., Puyraveau, M., Soumagne, T., Guillot, S., Rannou, F., Marquette, D., Berger, P., Jouneau, S., Monnet, E., Mauny, F., Laplante, J.-J., Dalphin, J.-C., & Degano, B. (2016). Prevalence and risk factors for COPD in farmers: A cross-sectional controlled study. The European Respiratory Journal, 47(1), 95–103. Ovid MEDLINE(R) <2016>. https://doi.org/10.1183/13993003.00153-2015	Respiratory
Hagen et al.	2019	Hagen, Briana N M, Albright, Ashley, Sargeant, Jan et al.. Research trends in farmers' mental health: A scoping review of mental health outcomes and interventions among farming populations worldwide.. PloS one, 2019, 14	Mental Health
Hagen et al.	2020	Hagen, B. N. M., Winder, C. B., Wootten, J., McMullen, C. K., & Jones-Bitton, A. (2020). A Systematic Review and Meta-Analysis of Depression among Farming Populations Worldwide. International Journal of Environmental Research and Public Health, 17(24). https://doi.org/10.3390/ijerph17249376	Mental Health
Hurwitz et al.	2023	Hurwitz, L. M., Freeman, L. E. B., Andreotti, G., Hofmann, J. N., Parks, C. G., Sandler, D. P., ... & Koutros, S. (2023). Joint associations between established genetic susceptibility loci, pesticide exposures, and risk of prostate cancer. Environmental Research, 237, 117063.	Cancer
Islam et al.	2022	Islam, J. Y., Mohamed, A., Umbach, D. M., London, S. J., Henneberger, P. K., Freeman, L. E. B., ... & Hoppin, J. A. (2022). Allergic and non-allergic wheeze among farm women in the Agricultural Health Study (2005–2010). Occupational and environmental medicine, 79(11), 744-751.	Respiratory

Jain et al.	2018	Jain, Rahul, Meena, M L, Dangayach, G S. Prevalence and risk factors of musculoskeletal disorders among farmers involved in manual farm operations.. International journal of occupational and environmental health, 2018, : 1-6	Musculoskeletal
Jamal et al.	2016	Jamal, F., Haque, Q. S., & Singh, S. (2016). Interrelation of glycemic status and neuropsychiatric disturbances in farmers with organophosphorus pesticide toxicity. The open biochemistry journal, 10, 27.	Poisoning
Jayasekara	2019	Jayasekara, K. B., Kulasooriya, P. N., Wijayasiri, K. N., Rajapakse, E. D., Dulshika, D. S., Bandara, P., ... & Albert, S. M. (2019). Relevance of heat stress and dehydration to chronic kidney disease (CKDu) in Sri Lanka. Preventive medicine reports, 15, 100928.	Renal
Jouneau et al.	2022	Jouneau, S., Chapron, A., Ropars, C., Marette, S., Robert, A. M., Gouyet, T., ... & Verger, C. (2022). Prevalence and risk factors of asthma in dairy farmers: Ancillary analysis of AIRBAg. Environmental Research, 214, 114145.	Respiratory
Juntarawijit et al.	2018	Juntarawijit, C., & Juntarawijit, Y. (2018). Association between diabetes and pesticides: a case-control study among Thai farmers. Environmental Health and Preventive Medicine, 23, 1-10.	Diabetes
Kangkhetkron et al.	2020	Kangkhetkron, T., & Juntarawijit, C. (2020). Pesticide exposure and lung cancer risk: A case-control study in Nakhon Sawan, Thailand. F1000Research, 9.	Cancer
Kanika et al.	2021	Arora, Kanika, Xu, Lili, Bhagianadh, Divya. Dementia and cognitive decline in older adulthood: Are agricultural workers at greater risk?. The Journals of Gerontology: Series B: Psychological Sciences and Social Sciences, 2021, 76: 1629-1643	Neurological
Keogh et al.	2022	Keogh, S. A., Leibler, J. H., Sennett Decker, C. M., Amador Velázquez, J. J., Jarquin, E. R., Lopez-Pilarte, D., ... & Scammell, M. K. (2022). High prevalence of chronic kidney disease of unknown etiology among workers in the Mesoamerican Nephropathy Occupational Study. BMC nephrology, 23(1), 238.	Renal
Kiatkitroj et al.	2022	Kiatkitroj, K., Arphorn, S., Tangtong, C., Maruo, S. J., & Ishimaru, T. (2022). Risk factors associated with heat-related illness among sugarcane farmers in Thailand. Industrial Health, 60(5), 447–458. https://doi.org/10.2486/indhealth.2021-0161	Other Non-communicable disease
Kumaraveloo et al.	2018	Kumaraveloo, K Sakthiaseelan, Lunner Kolstrup, Christina. Agriculture and musculoskeletal disorders in low- and middle-income countries.. Journal of agromedicine, 2018, 23: 227-248	Musculoskeletal
Lee et al.	2020	Lee, S., Lee, H., Kim, H. S., & Koh, S. B. (2020). Incidence, risk factors, and prediction of myocardial infarction and stroke in farmers: a Korean nationwide population-based study. Journal of Preventive Medicine and Public Health, 53(5), 313.	Cardiovascular
Liu et al.	2015	Liu, Shuo, Chen, Donghong, Fu, Shuang et al.. Prevalence and risk factors for farmer's lung in greenhouse farmers: an epidemiological study of 5,880 farmers from Northeast China.. Cell biochemistry and biophysics, 2015, 71: 1051-7	Respiratory
Lopez-Moreno et al.	2022	Lopez-Moreno, G., Davies, P., Yang, M., Culhane, M. R., Corzo, C. A., Li, C., Rendahl, A., & Torremorell, M. (2022). Evidence of influenza A infection and risk of transmission between pigs and farmworkers. Zoonoses and Public Health, 69(5), 560–571. https://doi.org/10.1111/zph.12948	Any Communicable disease
Lucero et al.	2021	Lucero, B., & Muñoz-Quezada, M. T. (2021). Neurobehavioral, neuromotor, and neurocognitive effects in agricultural workers and their children exposed to pyrethroid pesticides: a review. Frontiers in Human Neuroscience, 15, 648171.	Neurological
Martinot et al.	2023	Martinot, A., Adenis, A., Brousse, P., Govindin, Y., Rousseau, C., Thomas, N., ... & Bonifay, T. (2023). Cardiovascular Risk Assessment among Farmers in French Guiana in 2018—A Screening Program. International Journal of Environmental Research and Public Health, 20(2), 1262.	Cardiovascular
Montgomery et al.	2024	Montgomery, A., Basey, S., Baucom, L., & Scoggins, C. (2024). Stress and suicidal ideation among first-generation farmers: A cross-sectional study with 1,288 farmers in Georgia. The Journal of Rural Health, 40(1), 75–86. https://doi.org/10.1111/jrh.12764	
Moyce et al.	2019	Moyce, S., Hernandez, K., & Schenker, M. (2019). Diagnosed and undiagnosed diabetes among agricultural workers in California. Journal of health care for the poor and underserved, 30(4), 1289-1301.	Diabetes

Moyce et al.	2017	Moyce, Sally, Mitchell, Diane, Vega, Alondra et al.. Hydration Choices, Sugary Beverages, and Kidney Injury in Agricultural Workers in California.. Journal of nursing scholarship : an official publication of Sigma Theta Tau International Honor Society of Nursing, 2020, 52: 369-378	Renal
Muniswamy et al.	2021	Muniswamy, Sundar, Maliakel, Steffi F. A Comparative Study on the Health Problems and Substance Abuse among the Tobacco Farmers and Non-Tobacco Farmers in Hassan District, Karnataka.. Indian journal of occupational and environmental medicine, 2021, 25: 33-38	Substance use
Nankongnab et al.	2019	Nankongnab, Noppanun, Kongtip, Pornpimol, Tipayamongkholgul, Mathuros et al.. Difference in Accidents, Health Symptoms, and Ergonomic Problems between Conventional Farmers Using Pesticides and Organic Farmers.. Journal of agromedicine, 2020, 25: 158-165	Multi-topic
Nuvey et al.	2023	Nuvey, Francis Sena, Haydon, Daniel T, Hattendorf, Jan et al.. Relationship between animal health and livestock farmers' wellbeing in Ghana: beyond zoonoses.. BMC public health, 2023, 23	Other health outcome
O'Connor et al.	2024	O'Connor, Siobhan, Malone, Sandra M, Firnhaber, Joseph et al.. Disordered alcohol and substance use in Irish farmers: A cross-sectional survey.. The Journal of rural health : official journal of the American Rural Health Association and the National Rural Health Care Asso, 2024, 40: 173-180	Substance use
Olson et al.	2023	Olson, R. M., Nolan, C. P., Limaye, N., Osei, M., & Palazuelos, D. (2023). National Prevalence of Diabetes and Barriers to Care Among US Farmworkers and Association With Migrant Worker Status. Diabetes Care, 46(12), 2188-2192.	Diabetes
Petit et al.	2023	Petit, P., Gandon, G., Dubuc, M., Vuillerme, N., & Bonneterre, V. (2023). Agricultural activities and risk of treatment for depressive disorders among the entire French agricultural workforce: the TRACTOR project, a nationwide retrospective cohort study. The Lancet Regional Health–Europe, 31.	Mental Health
Petit et al.	2024	Petit, P., Leroyer, A., Chamot, S., Fumery, M., & Bonneterre, V. (2024). Farming activities and risk of inflammatory bowel disease: A French nationwide population-based cohort study. Journal of Crohn’s & Colitis, 101318676. https://doi.org/10.1093/ecco-jcc/jjae050	Other Non-communicable disease
Petit et al.	2024	Petit, P., Leroyer, A., Chamot, S., Fumery, M., & Bonneterre, V. (2024). Farming activities and risk of inflammatory bowel disease: A French nationwide population-based cohort study. Journal of Crohn’s & Colitis, 101318676. https://doi.org/10.1093/ecco-jcc/jjae050	Other Non-communicable disease
Pinidiyapathirage et al.	2018	Pinidiyapathirage, J., O’Shannessy, M., Harte, J., Brumby, S., & Kitchener, S. (2018). Chronic disease and health risk behaviors among rural agricultural workforce in Queensland. Journal of agromedicine, 23(1), 32-39.	Other Non-communicable disease
Pinidiyapathirage et al.	2022	Pinidiyapathirage, J., Kitchener, S., McNamee, S., Wynter, S., Langford, J., Doyle, A., & McMahon, A. (2019). Analysis of agriculture-related life-threatening injuries presenting to emergency departments of rural generalist hospitals in Southern Queensland. Emergency Medicine Australasia : EMA, 31(4), 587–592. https://doi.org/10.1111/1742-6723.13215	Accident
Quansah et al.	2016	Quansah, Reginald, Bend, John R, Abdul-Rahaman, Abukari et al.. Associations between pesticide use and respiratory symptoms: A cross-sectional study in Southern Ghana.. Environmental research, 2016, 150: 245-254	Respiratory
Quansah et al.	2019	Quansah, Reginald, Bend, John R, Armah, Frederick Ato et al.. Respiratory and non-respiratory symptoms associated with pesticide management practices among farmers in Ghana's most important vegetable hub.. Environmental monitoring and assessment, 2019, 191	Respiratory
Renier et al.	2024	Renier, M., Hippert, J., Louis-Bastien, W., Tual, S., Meryet-Figuiere, M., Vigneron, N., ... & Lebailly, P. (2024). Agricultural exposure and risk of ovarian cancer in the AGRIculture and CANcer (AGRICAN) cohort. Occupational and Environmental Medicine, 81(2), 75-83.	Cancer
Rosa et al.	2024	Rosa, M. J., Armendariz-Arnez, C., Gudayol-Ferre, E., Prehn, M., Fuhrimann, S., Eskenazi, B., Lindh, C. H., & Mora, A. M. (2024). Association of pesticide exposure with neurobehavioral outcomes among avocado farmworkers in Mexico. International Journal of Hygiene and Environmental Health, 256(do6, 100898843), 114322. Ovid MEDLINE(R). https://doi.org/10.1016/j.ijheh.2024.114322	Neurological
Sankoh et al.	2016	Sankoh, Alhaji I, Whittle, Rebecca, Semple, Kirk T et al.. An assessment of the impacts of pesticide use on the environment and health of rice farmers in Sierra Leone.. Environment international, 2016, 94: 458-466	Poisoning

Sato et al.	2020	Sato, Miho, Kato, Hiromi, Noguchi, Makiko et al.. Gender Differences in Depressive Symptoms and Work Environment Factors among Dairy Farmers in Japan.. International journal of environmental research and public health, 2020, 17	Mental Health
Sharifirad et al.	2022	Sharifirad, M., Poursaeed, A., Lashgarara, F., & Mirdamadi, S. M. (2022). Risk factors for musculoskeletal problems in paddy field workers in northern iran: A community-based study. Journal of Research in Medical Sciences, 27(1), 77.	Musculoskeletal
Shearer et al.	2021	Shearer, J. J., Sandler, D. P., Andreotti, G., Murata, K., Shrestha, S., Parks, C. G., Liu, D., Alavanja, M. C., Landgren, O., Beane Freeman, L. E., & Hofmann, J. N. (2021). Pesticide use and kidney function among farmers in the Biomarkers of Exposure and Effect in Agriculture study. Environmental Research, 199(ei2, 0147621), 111276. https://doi.org/10.1016/j.envres.2021.111276	Renal
Shivakumar et al.	2024	Shivakumar, Mrithula, Welsh, Victoria, Bajpai, Ram et al.. Musculoskeletal disorders and pain in agricultural workers in Low- and Middle-Income Countries: a systematic review and meta-analysis.. Rheumatology international, 2024, 44: 235-247	Musculoskeletal
Sørensen et al.	2020	Sorensen, Tina B, Matsuzaki, Mika, Gregson, John et al.. Is agricultural engagement associated with lower incidence or prevalence of cardiovascular diseases and cardiovascular disease risk factors? A systematic review of observational studies from low- and middle-income countries.. PloS one, 2020, 15	Cardiovascular
Soto et al.	2022	Soto, S., Yoder, A. M., Nuño, T., Aceves, B., Sepulveda, R., & Rosales, C. B. (2022). Health conditions among farmworkers in the Southwest: An analysis of the National Agricultural Workers Survey. Frontiers in Public Health, 10, 962085.	Multi-topic
Steen et al.	2023	Steen, Natalie A, Krokstad, Steinar, Torske, Magnhild Oust. A Cross-Sectional Study of Farmer Health and Wellbeing in Norway: The HUNT Study (2017-2019).. Journal of agromedicine, 2023, 28: 809-820	Multi-topic
Van Doorn et al.	2018	van Doorn, D, Richardson, N, Storey, A et al.. Farming characteristics and self-reported health outcomes of Irish farmers.. Occupational medicine (Oxford, England), 2018, 68: 199-202	Multi-topic
Watanabe-Galloway et al.	2022	Watanabe-Galloway, S., Chasek, C., Yoder, A. M., & Bell, J. E. (2022). Substance use disorders in the farming population: Scoping review. The Journal of Rural Health, 38(1), 129–150. https://doi.org/10.1111/jrh.12575	Substance use
Wyss et al.	2022	Wyss, A. B., Hoang, T. T., Vindenes, H. K., White, J. D., Sikdar, S., Richards, M., ... & London, S. J. (2022). Early-life farm exposures and eczema among adults in the Agricultural Lung Health Study. Journal of Allergy and Clinical Immunology: Global, 1(4), 248-256.	Other Non-communicable disease
Yamasaki et al.	2020	Yamasaki, M., Yano, S., Takeda, M., Abe, T., Isomura, M., Hamano, T., ... & Shiwaku, K. (2020). Prevalence of lifestyle-related chronic diseases among agricultural and non-agricultural workers in ruralareas of Japan: the Shimane CoHRE study. Journal of Rural Medicine, 15(1), 1-7.	Cardiovascular
Zhang et al.	2017	Zhang, S., Liu, Z., Liu, Y. L., Wang, Y. L., Liu, T., & Cui, X. B. (2017). Prevalence of stroke and associated risk factors among middle-aged and older farmers in western China. Environmental health and preventive medicine, 22, 1-6.	Cardiovascular
