# Supplementary material for: Unequal harvests: AI-assisted evidence map of trends and gaps in global farmer health research along SDG 3 priorities
Source: BMJ Open. 2026 Jun 1;16(6):e110537. doi: 10.1136/bmjopen-2025-110537 (PMC13239455; doi:10.1136/bmjopen-2025-110537)
Supplement: online supplemental file 3 [file bmjopen-16-6-s003.pdf]

Supplementary File S3. 2025 Sensitivity Screening Update (Bounded Sample)

Rationale
The main evidence map includes records published from 2015 through 19 June 2024. To address editorial concerns about whether newly published studies might change the overall patterns observed, we conducted a bounded sensitivity screening exercise using records published in 2025. This exercise was designed to identify the emergence of any **new dominant topic areas** or a **major shift** in the distribution of topics and income-setting patterns, rather than to provide a full update of the evidence map.

Search and sampling
We ran the same search strategy as described in the main manuscript (Supplementary Materials S1), restricted to publication year **2025**. The 2025 search, executed on February 11, 2026 retrieved **4,137** records. From these, we randomly selected **500** records for title and abstract screening.

Screening, inclusion, and coding
Screening and coding followed the same approach as the main evidence map. Titles and abstracts were used for screening and data extraction; **no full texts were used**. Records were screened using a single-screener approach. Included records were assigned (i) a health outcome category/tag and (ii) an income-setting category based on information available in the title/abstract and bibliographic metadata:

- **Health outcomes:** Records were tagged using the same outcome categories and definitions as the main manuscript (Supplementary Materials S3). Records reporting multiple outcomes could be assigned multiple tags.

- **Income-setting/Geography:** Where geographic information was available in the title/abstract, records were classified as **HIC** or **LMIC** using the World Bank 2023 income group classification (as in the main manuscript). If geographic location was not reported in the title/abstract, the record was coded as **"missing/global"(7 studies)** (depending on how the record was framed).

Ambiguous cases were resolved using the same decision rules applied in the main review.

Results
Of the 500 randomly selected 2025 records screened, **42** met the inclusion criteria and were coded. Among included records with any geographic information (n=35), the income-setting split was **43% HIC** and **57% LMIC**. The most frequently represented study location was the **USA (7/42)**, followed by **India (4/42)** and **China (4/42)** (based on country information reported in titles/abstracts; multi-country studies were counted for each named country in country-level tallies).

Interpretation
Because this is a bounded sample (42 included records) rather than a full update of the evidence map, findings should be interpreted as a **sensitivity check** only. We assessed whether the 2025 sample suggested (i) the emergence of a **new dominant health outcome domain** not observed in the main 2015–2024 evidence map, or (ii) a **reversal** of the main income-setting patterns. Neither was observed in this sample. While acknowledging that this sample is not powered to detect small percentage shifts, the distribution of outcome domains in 2025 remained broadly consistent with the main evidence map: injuries and mental health remained prominent, and LMIC records continued to include substantial work on pesticide poisoning/occupational exposures.

Table X1. Summary of included records in 2025 bounded sample (n=42)

	All studies*		HIC**		LMIC**	
	Overall number of studies	Percent age of Studies	Number of records with country information	Percent age of Studies	Number of records with country information	Percent age of Studies
All categories***	42	100%	15	43%	20	57%
**Non-communicable diseases**	7	**17%**	6	**40%**	1	**5%**
**Accidents and injuries**	14	**33%**	6	**40%**	8	**40%**
**Communicable diseases**	7	**17%**	2	**13%**	5	**25%**
**Mental health**	8	**19%**	5	**33%**	3	**15%**
General health and wellbeing	6	**14%**	3	**20%**	3	**15%**
Other health outcomes	1	**2%**	1	**7%**	0	**0%**

*Notes: *Includes all included studies (n=42), including records with missing geographic information (n=2) and global/multi-country perspectives without a single country setting (n=5). **HIC/LMIC columns include only records with any country or region information (n=35). ***Totals across topic rows exceed 100% because records reporting multiple outcomes were coded in all relevant categories.*

Data availability

The screened 2025 sample dataset (n=500; including inclusion decisions and assigned tags) is provided as **Supplementary Dataset S7 (CSV).**
