# Supplementary material for: Unequal harvests: AI-assisted evidence map of trends and gaps in global farmer health research along SDG 3 priorities
Source: BMJ Open. 2026 Jun 1;16(6):e110537. doi: 10.1136/bmjopen-2025-110537 (PMC13239455; doi:10.1136/bmjopen-2025-110537)
Supplement: online supplemental file 5 [file bmjopen-16-6-s005.pdf]

Supplementary Materials S5: Health Outcome Categories with Definitions and Relevant SDG Indicator and GBD Category

Tag	Definition	Relevant SDG Indicator	Relevant GBD Level 1 category
1. Communicable Diseases		3.3 / 3.9.2	Communicable, maternal, neonatal and nutritional diseases
1.1 Zoonoses	Zoonoses, including vector-borne diseases, parasitic infections, tick-borne diseases, malaria, and zoonotic transmissions of viruses, such as avian influenza, hepatitis E, or covid-19	3.3 / 3.9.2	
1.2 MRSA	Methicillin resistant Staphylococcus aureus (MRSA) and other infections with multidrug resistant organisms	3.3 / 3.9.2	
1.3 All other Communicable Diseases	All other types of infectious disease, including human tuberculosis, AIDS/HIV, water-borne infections, viruses, covid-19 and neglected tropical diseases.	3.3 / 3.9.2	
2. All Accidents and injuries			
2.1 Accidents and injuries	All occupational or non-occupational injuries, including accidents, (sexual) assault, violence, and violent or accidental death. Intentional self-harm and suicide outcomes were classified in the mental health category, including when the reported method involved pesticide ingestion.	part of [road traffic deaths] No specific SDG, included in 3.4	Injuries
2.2 Musculoskeletal	Health outcomes that involve musculoskeletal problems, and pain related to them, such as arthritis, occupational lower back pain, or carpal tunnel syndrome.	No specific SDG, included in 3.4	NCDs
2.3 Unintentional (Pesticide) Poisoning	Unintentional acute or chronic pesticide poisoning, including any health symptoms **due** to poisoning or accidents with pesticides, if the article made the explicit connection.	Part of 3.9.3	Injuries
3. Non communicable diseases (NCD)			
3.1 Diet-related NCD and Cardiovascular disease	Any cardiovascular illness, such as high blood pressure, heart disease, stroke. This includes risk factors for cardiovascular disease, such as metabolic syndrome and obesity.	3.4.1	NCDs
3.2 Cancer	Any cancer, including lung cancer, and all pre-cancer or benign oncologic lesions and related outcomes.	3.4.1	NCDs
3.3 Diabetes	Diabetes, insulin resistance, prediabetes etc	3.4.1	NCDs
3.4 Kidney disease	Renal illnesses, such as chronic kidney disease of unknown origin, including subclinical renal injury, heat illness and all health symptoms relating to heat exposure and dehydration.	No specific SDG, included in 3.4	NCDs
3.5 Respiratory or Pulmonary	Any illness related to the respiratory system, which includes lungs, bronchi and trachea, such as asthma, chronic obstructive pulmonary disease (COPD), cystic	3.4.1 / 3.9.1	NCDs

	fibrosis, farmer's syndrome, sleep apnea, or occupational lung diseases.		
3.6 Neurological	Any outcome related to neurological health, such as cognitive impairment, dementia etc. including long-term neurological consequences of pesticide exposure.	No specific SDG, included in 3.4	NCDs
3.7 Other Non-communicable disease or health issue	Any other specific NCD or health outcome not included above, such as autoimmune conditions, thyroid problems, green tobacco sickness, reproductive illness, hearing loss, olfactory impairment, sunburn, gastro-intestinal symptoms, sleep problems, fatigue, headaches, etc., if not explicitly mentioned as symptoms of pesticide poisoning or in the context of mental health issues.	No specific SDG, included in 3.4	NCDs
4 Mental health and substance use			
4.1 Mental health	Any mental illness or disorder, including suicide and suicidal ideation, suicide attempts, including studies where the reported method of self-harm involved pesticide ingestion, stress, anxiety, and health symptoms explicitly related to mental health issues or burnout.	3.4./3.4.2	Injuries/NCDs
4.1 Substance use	Any substance use disorder, substance or alcohol abuse or problematic use.	3.5	NCDs
5. General health	All studies reporting general (physical) health, all-cause mortality or morbidity and general wellbeing or health-related quality of life, not closer specified	No specific SDG, included in 3.4	N/A
