# Supplementary material for: Unequal harvests: AI-assisted evidence map of trends and gaps in global farmer health research along SDG 3 priorities
Source: BMJ Open. 2026 Jun 1;16(6):e110537. doi: 10.1136/bmjopen-2025-110537 (PMC13239455; doi:10.1136/bmjopen-2025-110537)
Supplement: Supplementary file 8 [file bmjopen-2025-110537.reviewer_comments.pdf]

PEER REVIEW HISTORY

BMJ Open publishes all reviews undertaken for accepted manuscripts. Reviewers are asked to complete a checklist review form and are provided with free text boxes to elaborate on their assessment. These free text comments are reproduced below.

ARTICLE DETAILS

Title (Provisional)

Unequal Harvests: AI-assisted evidence map of Trends and Gaps in Global Farmer Health Research along SDG 3 Priorities

Authors

Jaggi, Lena; Falgas Bague, Irene; Wey, Hannah; Rüfli, Daniel; Viglietti, Paola; Fuhrimann, Samuel

---

VERSION 1 - REVIEW

**Reviewer**	**1**
**Name**	**Kokol, Peter**
**Affiliation**	**Univerza v Mariboru Fakulteta elektrotehniko racunalnistvo informatiko**
**Date**	**01-Dec-2025**
**COI**	**None**

The theme of this bibliometric study is interesting. While the descriptive analysis was performed mostly competently, the study lacks the proper and formal qualitative analysis of descriptive Additionally, there are several more serious weaknesses:

1. Literature review in the introduction doesn't cover the recent period 2024-2025, the same is true for the seed articles and the corpus of paper in general. The year 2025 has to be included in the analysis

2. The research gap is not clear, neither are the research questions?

Furthermore, authors should delve into a deeper analysis of the research findings and go beyond reporting the quantitative trends. They should provide a more critical assessment of the the analysis output.

**Reviewer**	**2**
**Name**	**McCallum, Alison**

Affiliation **The University of Edinburgh Usher Institute of Population Health Sciences and Informatics, Centre for Population Health Sciences**

Date **12-Jan-2026**

COI **I am linked with the Edinburgh Evidence Synthesis Network which includes projects using Edinburgh Large Language Models and testing AI tools for evidence review and synthesis although I have not yet undertaken any projects using the tools being tested or the tool used in this review.**

General comments

This is a clearly written paper on an important topic. There are some significant omissions, both in the content of the paper and the supplementary files which will need to be addressed before it is suitable for publication.

Introduction

I felt the objectives and focus of the paper could be more explicit. It would be helpful for the authors to be clear whether their immediate focus is a rapid overview of maps and gaps to help advance the discourse, research, and policy agenda in a) occupational health for farmers and farm workers, b) regulation of working conditions or c) health systems that work for farmers and farm workers or all three. This would also enable them to link future work to learning from studies of health in populations where marginal, subsistence farming occurs and/or where people undertake farming and agricultural work as one of multiple occupations.

Methods

I would consider this a research record review rather than a full rapid review. There is no full text analysis. The inclusion of preliminary communications such as conference proceedings is a potential strength in an under-researched field, particularly one in evidence of effective interventions is emerging and has the potential to be globally important. However, reporting is incomplete. This could be remedied by amending the text to be explicit about where guidance was followed, where alternative measures were put in place and documenting the details in an expanded set of supplementary files.

Search strategy

Databases used

The abstract, paper and figures differ in their reporting of the databases used and this should be addressed. I appreciate that this is likely to be a simple drafting error but it meant that I was not clear whether Pubmed or Ovid Medline or both were used. Pubmed is often used in rapid reviews because it does not require an institutional licence to access all the features and tends to be a bit more sensitive to connection quality so it is often used instead

of Ovid Medline in some settings or simply for preliminary searches. If this is the case then this should be stated explicitly.

Classification

The rationale for using the IME Global Burden of Disease classification, rather than e.g. ICD chapter or causes of preventable morbidity and mortality, was not explicit. The rationale for classifying poisoning as intentional and unintentional where adequate data are available is helpful in terms of identifying the range of effective interventions required but its appropriateness in this study is not stated explicitly, particularly given the risk of misattribution.

The treatment of multi-country regions was not clear, particularly in relation to cross-border workers or where farming, herding, and related practices do not always adhere to administrative boundaries.

The use of ILO data was a strength.

Planned reporting of methods

Although the authors describe some of the differences between what was done and a standard systematic review, it is not clear that the authors have taken account of the interim guidance on rapid reviews from Cochrane or the associated publications as these were not included in the references (see for example Devane D, Hamel C, Gartlehner G, Nussbaumer-Streit B, Griebler U, Affengruber L, Saif-Ur-Rahman KM, Garritty C. Key concepts in rapid reviews: an overview. Journal of Clinical Epidemiology 2024; 175:111518. https://doi.org/10.1016/j.jclinepi.2024.111518. (https://www.sciencedirect.com/science/article/pii/S0895435624002749) or the associated series in BMJ Evidence Based Medicine Rapid Reviews Methods Series | BMJ Evidence-Based Medicine, particularly Stevens A, Hersi M, Garritty C, et al. BMJ Evidence-Based Medicine 2025;30:118–123. 10.1136/bmjebm-2024-112899.

Since the focus of the review is abstracts, a table that demonstrates reporting in line with the abstract reporting guidance in PRISMA 2020 explanation and elaboration: updated guidance and exemplars for reporting systematic reviews | The BMJ would have been helpful.

Relative weighting of evidence from reviews versus individual studies and avoidance of double counting.

It would have been help to have an explicit statement about how this was addressed.

Replication of findings

I could not replicate the findings using the search strategy and syntax provided and recommend that a copy of the search and syntax is included for one of the search engines used. However, when I used modified syntax and set limits similar to those reported by the authors, the number of studies returned was of the same magnitude.

I found few relevant abstracts in the first 500 results and I can see that this makes a good case for automating screening.

Results

A table summarising the studies included would be really helpful. This also provides transparency regarding the data used in the analysis (the included studies) and the findings (largely map and gaps) summarised in the paper.

Discussion

The study starts to set a research agenda for action and this is its main strength. It recognises that questions of gender, ethnicity, socio-economic status, and power imbalances could not be addressed based on the available data.

I felt the evidence for the research themes and links to the wider determinants of health could have been strengthened. In making the case for including reducing occupation related harm within the relevant SDG and developing indicators to help monitor progress towards farm workers' rights to health.

Limitations of the study

The limitations of automated screening tools, and research undertaken on this topic are not covered in any detail. Many groups use tools like COVIDENCE as a reference and review management tool to facilitate collaboration and coordination of the review but not to replace human decision making.

Ethics and due diligence

The nature of the institutional contract with the company that makes the active screener tool is not described. This should be stated explicitly as it is not clear whether matters of copyright and consent have been addressed with authors, journals, and institutions responsible for the papers that were used to train the active screening tool.

The tool is owned by a company that also receives funding from British American Tobacco (and other unhealthy commodity industries from which institutions may have divestment plans that this may be seen to compromise).

VERSION 1 - AUTHOR RESPONSE

Reviewer: 1

1. **Comment: "The theme of this bibliometric study is interesting. While the descriptive analysis was performed mostly competently, the study lacks the proper and formal qualitative analysis of descriptive. Additionally, there are several more serious weaknesses:"**

Response:

We thank the Reviewer for these comments. We have revised the manuscript to strengthen

the framing, explicitly state the mapping research questions, and expand the Discussion to provide a more critical interpretation of observed trends and inequities (policy and research agenda implications), while remaining consistent with an evidence-mapping design.

2. **Comment: Literature review in the introduction doesn't cover the recent period 2024-2025, the same is true for the seed articles and the corpus of paper in general. The year 2025 has to be included in the analysis**

Response:

Thank you for this comment. We have clarified the **search execution date** and analytic window (records published from **January 2015 through 19 June 2024**), which reflects the SDG-era period covered by our evidence map. The Introduction is intentionally focused on the **substantive gap** in global farmer health research and equity (including ILO workforce estimates that remain consistent with the period mapped) rather than providing a comprehensive year-by-year literature review. We also note that we do cite **recent sources (including 2025 where relevant)** to contextualise the contribution, but the paper's core results necessarily reflect the literature up to the prespecified search date.

Because the workflow relies on AI-assisted prioritisation and a stopping rule based on recall estimation within a fixed retrieved dataset, a full 2025 update would require re-running the search, de-duplication, and screening workflow on the expanded corpus and is beyond the scope of this revision. To address the request for 2025 coverage in a feasible way, we conducted a **bounded 2025 sensitivity screening, as outlined in the responses to the editor above.** This sensitivity check did not indicate new dominant topic areas or a reversal of the major geographic/income-setting patterns observed in the main 2015–2024 evidence map; however, we note that it is a sensitivity check rather than a full update.

3. **Comment: The research gap is not clear, neither are the research questions?**

Response :

In the revised manuscript, we have reframed the study as an **AI-assisted evidence map of research records**, and we have updated the aims accordingly to reflect the mapping purpose (research activity and gaps rather than synthesis of findings). We now present a clearly signposted, numbered set of aims to guide the reader, covering (1) geographic distribution by income group, (2) topical coverage across major outcome domains, and (3) coverage and gaps relative to SDG 3 targets, which we hope addresses the concern regarding clarity of the study focus and guiding questions.

4. **Comment: "authors should delve into a deeper analysis of the research findings and go beyond reporting the quantitative trends. They should provide a more critical assessment of the analysis output."**

Response :

Thank you for this comment. We revised the Discussion and Conclusions to go beyond reporting descriptive distributions by adding a more interpretive and critical assessment of what the mapped patterns imply. In particular, we now explicitly discuss how **differences in topic focus and geographic coverage likely reflect structural drivers**, such as surveillance capacity, occupational health reporting systems, regulatory protections, and research infrastructure, rather than burden alone. We also strengthened the implications of the findings by **highlighting the mismatch between where agricultural workers are concentrated and where research is produced**, and by clarifying how gaps in SDG 3 targets/indicators for key occupational risks (e.g., injuries, musculoskeletal conditions, heat-

related illness) limit accountability and policy action. We also **strengthened the Limitations** to clarify what inferences can and cannot be drawn from abstract-level mapping. These additions provide a more critical interpretation of the evidence-map outputs while remaining consistent with an abstract-based mapping design.

Reviewer: 2

General comments: "This is a clearly written paper on an important topic. There are some significant omissions, both in the content of the paper and the supplementary files which will need to be addressed before it is suitable for publication."

Response:
We thank the Reviewer for these comments. We addressed the omissions by expanding the supplementary materials (full search strategies; PRISMA-ScR checklist; and additional transparency outputs) and revising the Methods and Discussion to better match an evidence-mapping design.

5. **Introduction comment: "I felt the objectives and focus of the paper could be more explicit. It would be helpful for the authors to be clear whether their immediate focus is a rapid overview of maps and gaps to help advance the discourse, research, and policy agenda in a) occupational health for farmers and farm workers, b) regulation of working conditions or c) health systems that work for farmers and farm workers or all three. This would also enable them to link future work to learning from studies of health in populations where marginal, subsistence farming occurs and/or where people undertake farming and agricultural work as one of multiple occupations."**

Response :
To make the objectives and focus more explicit, we revised the Introduction to include a clearly signposted aims paragraph beginning: **"To address this gap, we conducted an AI-assisted evidence map of research records…"**, followed by three numbered aims covering (1) geographic distribution by income group, (2) topical coverage across major outcome domains, and (3) coverage and gaps relative to SDG 3 priorities. We believe these revisions clarify that the paper's purpose is to map research activity and gaps to inform a broad research and policy agenda for farming populations. See also our answer to the editors comment #3 above. Finally, we added the following to the limitations section: "This may be particularly relevant in settings where farming is subsistence-based or one of multiple occupations, and where agricultural work and where informal agricultural work is common."

6. **Methods/design comment: "I would consider this a research record review rather than a full rapid review. There is no full text analysis. The inclusion of preliminary communications such as conference proceedings is a potential strength in an under-researched field, particularly one in evidence of effective interventions is emerging and has the potential to be globally important. However, reporting is incomplete. This could be remedied by amending the text to be explicit about where guidance was followed, where alternative measures were put in place and documenting the details in an expanded set of supplementary files."**

Response :
We agree and have reframed the manuscript as an **AI-assisted evidence map of research records** based on titles/abstracts and bibliographic metadata rather than a rapid review of study findings. We thoroughly revised the Methods and Data Extraction sections to be explicit that (i) screening and coding were performed using **titles/abstracts only** (no full-text extraction), (ii) the objective is to map **research activity and gaps** rather than synthesise findings or estimate effects, and (iii) we used an **AI-assisted prioritisation tool** to support screening order while maintaining **human-only inclusion decisions and coding**, with team meetings to refine decision rules and adjudicate borderline cases. We have also aligned reporting with **PRISMA-ScR** as the most appropriate guidance for this scoping-style mapping review and included the completed PRISMA-ScR checklist in the appendix. Please see also our answer to your other comments below.

7. **Search strategy comment: "The abstract, paper and figures differ in their reporting of the databases used and this should be addressed. I appreciate that this is likely to be a simple drafting error but it meant that I was not clear whether Pubmed or Ovid Medline or both were used."**

Response :
Thank you, this was indeed a drafting error. We confirm we used Ovid/Medline (not PubMed) and have corrected the abstract, main text, figures, and supplementary files for consistency.

8. **Classification rationale comment: "The rationale for using the IME Global Burden of Disease classification… was not explicit."**

Response:
We added an explicit rationale for using the Global Burden of Disease-aligned grouping as a pragmatic framework that enables consistent categorisation across diverse conditions and facilitates mapping to SDG 3 targets. Specifically, we added the following to the Data Extraction section: *"We used the GBD level 1 structure as a pragmatic, widely used framework to support consistent categorisation across a diverse and multidisciplinary literature and to facilitate mapping of topics to SDG 3 priorities."*

9. **Poisoning classification comment: "The rationale for classifying poisoning as intentional and unintentional where adequate data are available is helpful in terms of identifying the range of effective interventions required but its appropriateness in this study is not stated explicitly, particularly given the risk of misattribution"**

Response :
We distinguished publications describing **pesticide ingestion as a method of intentional self-harm/suicide** (classified under *mental health*) from publications describing **unintentional pesticide poisoning** from an occupational exposure/workplace safety perspective (classified under *injuries/accidents*). We applied the framing used by each record (based on title/abstract wording) and assigned categories accordingly when intent was explicitly stated; all inclusion and category assignments were made **manually by the review team**. We clarified this classification approach in the Methods and in the outcome definitions (Supplementary Materials S3). Specifically, we added the following to the Data Extraction section: "*For pesticide-related poisoning, we coded records describing pesticide ingestion as intentional self-harm/suicide under mental health, and records describing unintentional pesticide poisoning or occupational exposure under accidents and injuries,*

based on the framing and explicit wording in the title/abstract." and updated the definitions in Supplementary Materials S3.

10. **Multi-country regions comment: "The treatment of multi-country regions was not clear, particularly in relation to cross-border workers or where farming, herding, and related practices do not always adhere to administrative boundaries."**

Response:
Our geographic coding was based on information available in the **title and abstract** of each record. Thank you. Geographic coding in this evidence map reflects **where the study was conducted** as reported in the title/abstract (i.e., the location of the research), rather than the place of origin of study participants (e.g., studies of migrant farmworkers in California were coded as USA). We agree that cross-border mobility and non-administrative geographic boundaries are important considerations, but re-adjudicating how primary studies handled these issues was beyond the scope of this abstract-based evidence map. To improve clarity, we added the following in the Data Extraction section:

"Geographical location was extracted as reported in the title and abstract and reflects the location where the study was conducted (i.e., the geographic location of the research), not the participants' place of origin. We did not reclassify cross-border or non-administrative populations beyond what was stated in the record."

11. **Rapid review guidance comment: "Although the authors describe some of the differences between what was done and a standard systematic review, it is not clear that the authors have taken account of the interim guidance on rapid reviews from Cochrane or the associated publications as these were not included in the references..."**

Response :
Thank you for this insightful point. In response, we have revised the manuscript to **avoid presenting the work as a rapid review of study findings** and instead describe it as an **AI-assisted evidence map of research records** based on titles/abstracts and bibliographic metadata. We therefore did not adopt rapid review guidance as the primary methodological frame. Instead, we aligned reporting with **PRISMA-ScR** as the most appropriate guidance for our mapping aims and abstract-level methods, and revised the manuscript accordingly to clearly distinguish this evidence map from a rapid review/systematic review of effects.

12. **Abstract reporting guidance comment: "Since the focus of the review is abstracts, a table that demonstrates reporting in line with the abstract reporting guidance in PRISMA 2020 explanation and elaboration..."**

Response :
Thank you. In the revised manuscript, we have **tightened the abstract and Methods** to be explicit that data extraction and coding were based on **titles/abstracts and bibliographic metadata only**, with **no full-text extraction**. To strengthen reporting transparency, we also include a completed **PRISMA-ScR checklist** (given the evidence map/scoping-style framing) and ensured that the existing supporting materials are clearly referenced and complete, including the **flow diagram**, the **full search strategy**, and the **health outcome category definitions** in the supplementary materials.

13. **Double counting comment: "Relative weighting of evidence from reviews versus individual studies and avoidance of double counting. It would have been help to have an explicit statement about how this was addressed"**

Response :
Thank you. In this evidence map, the **unit of analysis is the publication ('research record')** rather than the underlying body of evidence. We therefore **did not weight record types differently** (e.g., reviews versus primary studies) and did not attempt to adjust counts for potential overlap where primary studies may also be represented within review articles. As stated in the methods already, Studies reporting multiple outcomes were tagged for each health outcome separately. To clarify this further, we added the following sentence to the Data Extraction section:

"Because the unit of analysis was the publication record, we did not weight reviews differently from primary studies and did not adjust counts for overlapping underlying evidence across record types."

14. **Replication comment: "I could not replicate the findings using the search strategy and syntax provided and recommend that a copy of the search and syntax is included for one of the search engines used. However, when I used modified syntax and set limits similar to those reported by the authors, the number of studies returned was of the same magnitude. I found few relevant abstracts in the first 500 results and I can see that this makes a good case for automating screening."**

Response :
We apologise for the difficulty replicating the search. The strategy in Supplementary Materials S1 included the core query string and indicated the publication year restriction ("2015–current"), but we recognise that in Ovid this restriction is often applied through the **Limits** checkboxes and may not be reproducible if readers attempt to paste the strategy without applying the same limits. We have therefore **updated Supplementary Materials S1** to (i) present the search as a **numbered Ovid search history** (line-by-line), and (ii) specify the **exact limits used** in a way that can be reproduced (e.g., "Limits: yr=2015–2024; humans; [any other limits if used]"), as well as the search execution date.

15. **Results transparency comment: "A table summarising the studies included would be really helpful. This also provides transparency regarding the data used in the analysis (the included studies) and the findings (largely map and gaps) summarised in the paper."**

Response :
We agree. Rather than a long PDF table, we provide a machine-readable supplementary dataset (e.g., CSV/Excel) in **Supplementary Materials S5** listing included records and extracted fields (e.g., year, country/region, topic categories, income group classification), to improve transparency and usability.

16. **Discussion comment: I felt the evidence for the research themes and links to the wider determinants of health could have been strengthened. In making the case for including reducing occupation related harm within the relevant SDG and developing indicators to help monitor progress towards farm workers' rights to health.**

Response :

Thank you for this helpful suggestion. We have strengthened the Discussion to more clearly connect the mapped research themes to wider determinants of health affecting farming populations (including working conditions, regulatory protections, and access to health services), and to articulate the implications for SDG monitoring and accountability. In particular, we expanded the section discussing how several high-burden occupational risks for agricultural workers (e.g., heat-related illness, musculoskeletal conditions, and occupational injuries) are incompletely reflected in SDG 3 indicators, and we clarify how improved indicators could support monitoring of progress toward farm workers' rights to health. We also make clearer that the observed research patterns leave broader structural determinants (including working conditions, occupational protections, and access to services) comparatively underexplored, especially in LMIC settings where farming is a major livelihood. Finally, we thoroughly reworked the Conclusions and **added text outlining practical opportunities for action** (strengthening occupational health surveillance and reporting, integrating occupational health within health systems, and investing in research capacity and data generation in LMIC agricultural settings) consistent with the evidence-mapping scope of this paper.

17. **Limitations comment: "The limitations of automated screening tools, and research undertaken on this topic are not covered in any detail. Many groups use tools like COVIDENCE as a reference and review management tool to facilitate collaboration and coordination of the review but not to replace human decision making."**

Response :

Thank you for highlighting this important point. We agree that, although the original Methods section described our use of SWIFT-Active Screener (including the number of records manually screened and the stopping threshold based on estimated recall), the manuscript did not sufficiently explain the **implications and limitations** of using an active-learning prioritisation tool in an abstract-based evidence map.

We have therefore **thoroughly reworked the Methods section** to clarify the role of SWIFT-Active Screener as a **prioritisation tool** (influencing only the order in which records were presented for screening) rather than a decision-maker, very similar with how tools such as Covidence are often used to facilitate screening workflows. In fact, we chose SWIFT-Active Screener for its similarity with Covidence, but with an improved option of detailed topical tagging during the inclusion/exclusion screening of title and abstract. We explicitly state that **all inclusion/exclusion decisions and topic classifications were made manually by human reviewers** based on titles/abstracts, with regular team discussions and adjudication of borderline cases to support consistent application of decision rules. We also clarify why the stopping threshold used is appropriate for an **evidence map intended to identify broad trends and gaps** rather than to synthesise effects or draw conclusions about intervention effectiveness. In addition, we added a **new, dedicated limitation** explicitly addressing AI-assisted screening. Specifically, the limitations section now reads:

"*Screening was supported by SWIFT-Active Screener, which uses active learning to prioritize records for review and integrated recall estimation to support stopping decisions.31,32 The tool did not replace human judgement: it influenced only the order in which records were screened, while all inclusion decisions and topic classifications were made manually based on titles/abstracts. Nonetheless, active-learning prioritization combined with abstract-only*

screening may lead to residual under-ascertainment or misclassification, particularly for niche topics or poorly described abstracts. While we consider the estimated recall threshold of 0.94 of the tool appropriate for describing broad trends in an evidence map, we acknowledge that it does not guarantee complete capture of all relevant records. We also acknowledge potential concerns related to the vendor's reported financial links to British American Tobacco; however, the software could not influence results beyond screening order, since all coding decisions were made by the research team. Finally, because we mapped research records based on titles/abstracts without full-text extraction or critical appraisal, the findings describe the distribution of research activity and should not be interpreted as evidence of intervention effectiveness or causal relationships.""

Finally, please also see our answer to the Editor's comment #5.

18. **Ethics/due diligence comment: "The nature of the institutional contract with the company that makes the active screener tool is not described. This should be stated explicitly as it is not clear whether matters of copyright and consent have been addressed with authors, journals, and institutions responsible for the papers that were used to train the active screening tool."**

Response :
We clarified in the Methods section that the institutional contract was a **standard time-limited licence** for software access. We also clarify that **tool training and screening used only bibliographic record data** (titles/abstracts and metadata) accessed through the databases, and we did not upload or analyse paywalled full texts within the tool. Specifically, the section now reads:

"To ensure the algorithm's relevance and accuracy, bibliographic record information retrieved from the databases (titles, abstracts, keywords, and associated metadata) of 90 seed articles representing a broad spectrum of health outcomes and study designs in farming populations were uploaded (see Supplementary Materials S2). These seed records were used to 1) conduct sensitivity checks to validate the retrieved results and 2) train the learning models to identify relevant studies, using the same title/abstract information available during screening."

Please also see our answer to your previous comment #27 and the Editor's comment #1

19. **Unhealthy commodity industry funding comment: "The tool is owned by a company that also receives funding from British American Tobacco (and other unhealthy commodity industries from which institutions may have divestment plans that this may be seen to compromise)"**

Response :
We acknowledge this concern and have added it as a limitation, alongside safeguards described above. We reiterate that no authors have any relationship with the company and that the tool's role was limited to prioritising the order of human screening in the Competing Interest statement. Please also see our answer to your previous comment #27 and the Editor's comment #2

VERSION 2 - REVIEW

Reviewer 2

Name **McCallum, Alison**

Affiliation **The University of Edinburgh Usher Institute of Population Health Sciences and Informatics, Centre for Population Health Sciences**

Date **11-Mar-2026**

COI

Thank you for your thoughtful and comprehensive revisions. These have addressed my concerns -other than the funding sources for the overarching company mean that institutions should be very cautious about the wider implications for using their products. I did find a small number of typos and one sentence I thought could be clearer - listed here.

Page 9, line 24-25 - typo

This search resulted in 32,006 records. After deduplication, 31,503 unique records remained for review, of which we manually screened 8’533.

Page 9, line 52 typo

The most studied health outcome were non-communicable diseases (NCDs),

Page 12, line 24 typo

mental health farming populations

Page13, line 12-13 the meaning of this sentence is not clear

Illustrative, a significant share (31%) of mental health studies drew on U.S. farmer samples, where institutional surveillance and policy support this work.

Page 15, line 48-49 typo

4) Screening was supported by SWIFT-Active Screener, which uses active learning to prioritize records for review and integrated recall estimation to support stopping decisions.31,32

VERSION 2 - AUTHOR RESPONSE

Reviewer: 2

Thank you for your thoughtful and comprehensive revisions. These have addressed my concerns - other than the funding sources for the overarching company mean that institutions should be very cautious about the wider implications for using their products.

Response: Thank you very much for this comment, we are glad that we were able to address your concerns and comments through our revisions.

I did find a small number of typos and one sentence I thought could be clearer - listed here.

Page 9, line 24-25 - typo
This search resulted in 32,006 records. After deduplication, 31,503 unique records remained for

review, of which we manually screened 8’533.
Page 9, line 52 typo
The most studied health outcome were non-communicable diseases (NCDs),
Page 12, line 24 typo
mental health farming populations
Page13, line 12-13 the meaning of this sentence is not clear
Illustrative, a significant share (31%) of mental health studies drew on U.S. farmer samples, where institutional surveillance and policy support this work.
Page 15, line 48-49 typo
4) Screening was supported by SWIFT-Active Screener, which uses active learning to prioritize records for review and integrated recall estimation to support stopping decisions.31,32

Response: Thank you for these detailed comments. We have corrected all typos and amended the sentence to now read as follows: “*Illustrative, a significant share (31%) of mental health studies drew on farmer samples from the U.S., a country where institutional surveillance and policy might lend more support for this research topic.”*
